# A genetically defined signature of responsiveness to erlotinib in early-stage pancreatic cancer patients: Results from the CONKO-005 trial

**DOI:** 10.1016/j.ebiom.2021.103327

**Published:** 2021-04-13

**Authors:** K. Hoyer, R. Hablesreiter, Y. Inoue, K. Yoshida, F. Briest, F. Christen, N. Kakiuchi, T. Yoshizato, Y. Shiozawa, Y. Shiraishi, J.K. Striefler, S. Bischoff, P. Lohneis, H. Putter, O. Blau, U. Keilholz, L. Bullinger, U. Pelzer, M. Hummel, H. Riess, S. Ogawa, M. Sinn, F. Damm

**Affiliations:** a Department of Hematology, Oncology, and Tumor Immunology, Charité – Universitätsmedizin Berlin, corporate member of Freie Universität Berlin, Humboldt-Universität zu Berlin, and Berlin Institute of Health, Augustenburger Platz 1, Berlin 13353, Germany; b Department of Pathology and Tumor Biology, Graduate School of Medicine, Kyoto University, Kyoto, Japan; c Laboratory of DNA information Analysis, Human Genome Centre, Institute of Medical Science, The University of Tokyo, Tokyo, Japan; d Charité – Universitätsmedizin Berlin, corporate member of Freie Universität Berlin, Humboldt-Universität zu Berlin, and Berlin Institute of Health, Institute of Pathology, Berlin, Germany; e Institute of Pathology, University of Cologne, Cologne, Germany; f Department of Biomedical Data Sciences, Leiden University Medical Center, Leiden, the Netherlands; g Charité Comprehensive Cancer Center, Charité – Universitätsmedizin Berlin, corporate member of Freie Universität Berlin, Humboldt-Universität zu Berlin, and Berlin Institute of Health, Berlin, Germany; h Institute for the Advanced Study of Human Biology (WPI-ASHBi), Kyoto University, Kyoto, Japan; i Department of Medicine, Centre for Haematology and Regenerative Medicine, Karolinska Institute, Stockholm, Sweden; j Department of Oncology, Hematology and Bone Marrow Transplantation with Division of Pneumology, University Medical Center Hamburg-Eppendorf, Hamburg, Germany; k German Cancer Consortium (DKTK), partner site Berlin, Berlin, Germany; l German Cancer Research Center (DKFZ), Heidelberg, Germany

**Keywords:** Pancreatic cancer, Precision medicine, Erlotinib, *SMAD4*, *MAPK9*

## Abstract

**Background:**

high recurrence rates of up to 75% within 2 years in pancreatic ductal adenocarcinoma (PDAC) patients resected for cure indicate a high medical need for clinical prediction tools and patient specific treatment approaches. Addition of the EGFR inhibitor erlotinib to adjuvant chemotherapy failed to improve outcome but its efficacy in some patients warrants predictors of responsiveness.

**Patients and Methods:**

we analysed tumour samples from 293 R0-resected patients from the randomized, multicentre phase III CONKO-005 trial (gemcitabine ± erlotinib) with targeted sequencing, copy number, and RNA expression analyses.

**Findings:**

a total of 1086 mutations and 4157 copy-number aberrations (CNAs) with a mean of 17.9 /tumour were identified. Main pathways affected by genetic aberrations were the MAPK-pathway (99%), cell cycle control (92%), TGFβ signalling (77%), chromatin remodelling (71%), and the PI3K/AKT pathway (65%). Based on genetic signatures extracted with non-negative matrix factorization we could define five patient clusters, which differed in mutation patterns, gene expression profiles, and survival. In multivariable Cox regression analysis, *SMAD4* aberrations were identified as a negative prognostic marker in the gemcitabine arm, an effect that was counteracted when treated with erlotinib (DFS: HR=1.59, *p* *=* 0.016, and OS: HR = 1.67, *p* *=* 0.014). Integration of differential gene expression analysis established *SMAD4* alterations with low *MAPK9* expression (*n* = 91) as a predictive biomarker for longer DFS (HR=0.49; test for interaction, *p* *=* 0.02) and OS (HR = 0.32; test for interaction, *p* *=* 0.001).

**Interpretation:**

this study identified five biologically distinct patient clusters with different actionable lesions and unravelled a previously unappreciated association of *SMAD4* alteration status with erlotinib effectiveness. Confirmatory studies and mechanistic experiments are warranted to challenge the hypothesis that *SMAD4* status might guide addition of erlotinib treatment in early-stage PDAC patients.

**Research in context**

**Evidence before this study**

The EGFR inhibitor Erlotinib is currently the only FDA-approved kinase inhibitor for patients with advanced pancreatic cancer showing some improvement in OS and DFS. When added to gemcitabine in the adjuvant setting, erlotinib failed to improve outcome for patients with early-disease stages. Molecular predictors of response are largely unknown for EGFR inhibition in PDAC. Searching PubMed using the terms “precision medicine” or “personalized medicine” or “integrative genomic profiling” together with “pancreatic cancer”, we identified and reviewed pertinent articles published in English before May 1st 2020. We were able to identify mainly review and perspective articles. In addition, some articles applying real-time genomic characterization to guide subsequent treatment strategies by actionable lesions were found. However, these latter studies were exclusively performed in advanced disease stages and only a subset of patients received molecularly matched therapy. Therefore, to the best of our knowledge there has been no large study published yet applying integrative genomics to a R0-resected PDAC patient cohort treated within a large multicentre phase III trial.

**Added value of this study**

We demonstrate that integrative genomic profiling of PDAC tumour samples resected for cure is capable to define distinct genetic subgroups harbouring different actionable lesions. This approach led to the discovery of a frequent molecular phenotype – defined by genetic aberrations in *SMAD4* coupled with low mRNA expression of *MAPK9* – as a predictor of erlotinib response. We show that such datasets can be used as a powerful tool for precision medicine approaches, especially in light of growing multimodal treatment possibilities.

**Implications of all the available evidence**

Mounting evidence underscores the potential of precision medicine approaches in metastatic PDAC patients. Our study underlines the rationale that tumour-based molecular profiling for patients with pancreatic cancer should also be routinely performed in early disease stages.

Alt-text: Unlabelled box

## 1 Introduction

With advances in next generation sequencing technologies, our knowledge of the molecular background of most cancer types has increased tremendously. This has not only led to a better understanding of the mutational processes in cancer, but has paved the way for more patient specific treatment approaches. Because driver mutations are causative, drugs that target the function of resulting proteins can be therapeutic. For example, the treatment of *EGFR* mutated non-small-cell lung cancers with EGFR inhibitors [1,2] like erlotinib improved patients outcome significantly and is nowadays standard of care.

However, patients with pancreatic ductal adenocarcinoma (PDAC) have so far not benefited from recent improvements made by precision medicine approaches in other malignancies. Clinical outcome of PDAC remains dismal, with a 5-year survival rate below 10% across all stages and a median survival of <11 months in advanced disease. PDAC is the fourth leading cause of cancer-related death in Western societies [3]. Several large-scale sequencing studies revealed a complex mutational landscape underlying PDAC carcinogenesis with recurrent oncogenic events in four well-known cancer genes (*KRAS, TP53, SMAD4,* and *CDKN2A*), as well as a long list of rather infrequent alterations, and established RNA-based PDAC subtyping [4], [5], [6]. However, due to the lack of molecular targets and clear predictive factors, this current knowledge could not be transferred into relevant clinical decision making so far. Limited sample size, and heterogeneity within clinical trial cohorts might have masked the impact of genetic alterations.

To address this knowledge gap, we performed a molecular in-depth characterization of 293 R0-resected PDAC patients treated within the CONKO-005 trial, a randomized, multicentre phase III trial which compared adjuvant chemotherapy of gemcitabine with or without the tyrosine kinase inhibitor erlotinib [7]. While no significant difference in disease-free survival (DFS) and overall survival (OS) could be observed between the two treatment arms, our aim of this analysis was to identify patient subgroups with a potential benefit from additional erlotinib by integrative genomics using a combination of mutation, copy number, and gene expression analyses.

## 2 Methods

### 2.1 Patients

All patients were enrolled in the CONKO-005 study, an open-label, multicentre, randomized phase III trial investigating the addition of erlotinib to gemcitabine compared to gemcitabine only as adjuvant therapy [7]. Treatment details have been published and are summarized in the Supplemental Appendix. No differences with respect to baseline characteristics and patients’ outcome between the 293 patients from our study population and the entire CONKO-005 cohort was observed (Table 1 and Supplemental Fig. S1).

**Table 1:** Clinical baseline characteristics of 293 PDAC patients from the CONKO-005 trial.

Characteristics	PDAC
	(*n* = 293)
Age, years	
Median	64
Range	24–82
Sex	
male - no. (%)	163 (56%)
female - no. (%)	130 (44%)
Arm	
Gemcitabine - no. (%)	149 (51%)
Gemcitabine + Erlotnib - no. (%)	144 (49%)
Karnofsky	
60 - no. (%)	1 (>1%)
70 - no. (%)	10 (3%)
80 - no. (%)	75 (26%)
90 - no. (%)	112 (38%)
100 - no. (%)	95 (32%)
Grading	
G1 - no. (%)	7 (2%)
G2 - no. (%)	180 (61%)
G3 - no. (%)	96 (33%)
unknown - no. (%)	10 (4%)
T-Stage	
T1 - no. (%)	9 (3%)
T2 - no. (%)	30 (10%)
T3 - no. (%)	251 (86%)
T4 - no. (%)	3 (1%)
N-Stage	
N0 - no. (%)	106 (36%)
N1 - no. (%)	187 (64%)
Postoperative CA 19–9, kU/L	
Median (range)	19,5 (1–5816)
≤ 100 - no. (%)	223 (76%)
101–500 - no. (%)	29 (10%)
> 500 - no. (%)	12 (4%)
unknown - no. (%)	29 (10%)

### 2.2 Ethics approval

Written consent was obtained from every individual in accordance with the Declaration of Helsinki and with ethical approval obtained from the local ethics committee from the Charité – Universitätsmedizin Berlin, Germany (EAl/139/05, amendment 12.08.2012).

### 2.3 Mutation analysis by targeted sequencing

DNA and RNA were extracted from 331 FFPE samples, with a tumour content of at least 10% (Supplemental Table S1). The sequencing panel covered full-length coding regions of 67 genes described as significantly mutated [5,6], shown as clinically relevant [8], [9], [10], included in previous panels [9,11], and/or representing major players in the EGFR pathway (Supplemental Table S2). A custom Agilent SureSelectXT Target Enrichment System for Illumina Paired-End Multiplexed Sequencing was used and libraries were paired-end sequenced with a mean sequencing depth of ~600x and a minimal reading depth of 200x. Further information on filtering criteria are outlined in the Supplemental Appendix.

To reduce the likelihood of false single nucleotide variant (SNV) calling, we established a validation pipeline using a combination of SNV frequency, EBcall *p*-value, DNA quality, and sequencing duplication rate. A total of 219 potential SNVs representing 17% of all detected variants were investigated in a second independent experiment either by amplicon-based targeted deep sequencing (*n* = 195) or ddPCR (*n* = 24) as previously described [12], [13], [14]. With a mean coverage of 88102x, we could validate 210 variants, which led to a high validation rate of 96%.

### 2.4 Copy-number alterations (CNA) detection by targeted sequencing

Copy-number analysis was performed as previously reported using an in-house pipeline CNACS (https://github.com/papaemmelab/toil_cnacs) (Y. Shiozawa and S.Ogawa, manuscript in preparation) [15], in which total number of reads covering each bait region and allele frequency of heterozygous single-nucleotide polymorphisms (SNP) were used as input data (see also Supplemental Appendix and Table S3). For 171 patients we could detect CNAs based on targeted sequencing data. For validation and remaining 112 samples, we generated information about local CNA in 11 genes based solely on a Multiplex Ligation-dependant Probe Amplification (MLPA) assay. For 10 patients we were not able to obtain CNA data with either method (Supplemental Fig. S2).

### 2.5 Expression profiling with nCounter technology

The NanoString nCounter Flex system was used to run a customized version of the PanCancer Pathways Panel (770 genes representing 13 canonical pathways in cancer; Supplemental Appendix and Table S4). After RNA quality controls, 230 samples were analysed. Genes were tested for differential expression in response to each selected covariate. For each gene, a single linear regression was fit using all selected covariates to predict expression and false discovery rate (FDR) was estimated according to the Benjamini-Hochberg procedure [16].

### 2.6 Statistical analysis

Statistical analyses were performed using IBM SPSS statistics (version 24) and R (version 3.6.1) and are detailed in the Supplemental Appendix. Co-occurrence and mutational exclusivity was calculated with Fisher's exact test and subsequently corrected for multiple testing [16]. To model clonal composition we used a modified version of the *SciClone* Bioconductor package as previously described [17,18]. Signatures extracted from SNV and CNA patterns with non-negative matrix factorization (NMF) were used to group patients into five distinct clusters [19]. Step-wise subsampling of the patient cohort (minus five patients per step) was performed to ensure a high stability of the identified signatures and, therefore a high cluster stability (Supplemental Fig. S3). Cox models were used for time to-event variables (OS and DFS), and *p*-values were calculated using the Wald test. Multivariable cox proportional hazards models were used to investigate variables associated with survival endpoints. To select input for the multivariable cox proportional hazards models, univariate cox regression analysis of clinical and genetic variables were carried out. Primary analysis endpoint was OS, followed by exploratory analysis for DFS. Kaplan-Meier analysis was performed to construct survival curves and log-rank test was applied to evaluate differences between subgroups.

#### 2.6.1 Role of funding source

The funders of this study had no role in study design, data collection, data analysis, data interpretation, or writing of the report. KH, RH, IY, KY, MS, and FD had access to the raw data. FD had full access to all the data in the study and had final responsibility for the decision to submit for publication.

## 3 Results

### 3.1 Genetic landscape of PDAC

**Fig. 1:**
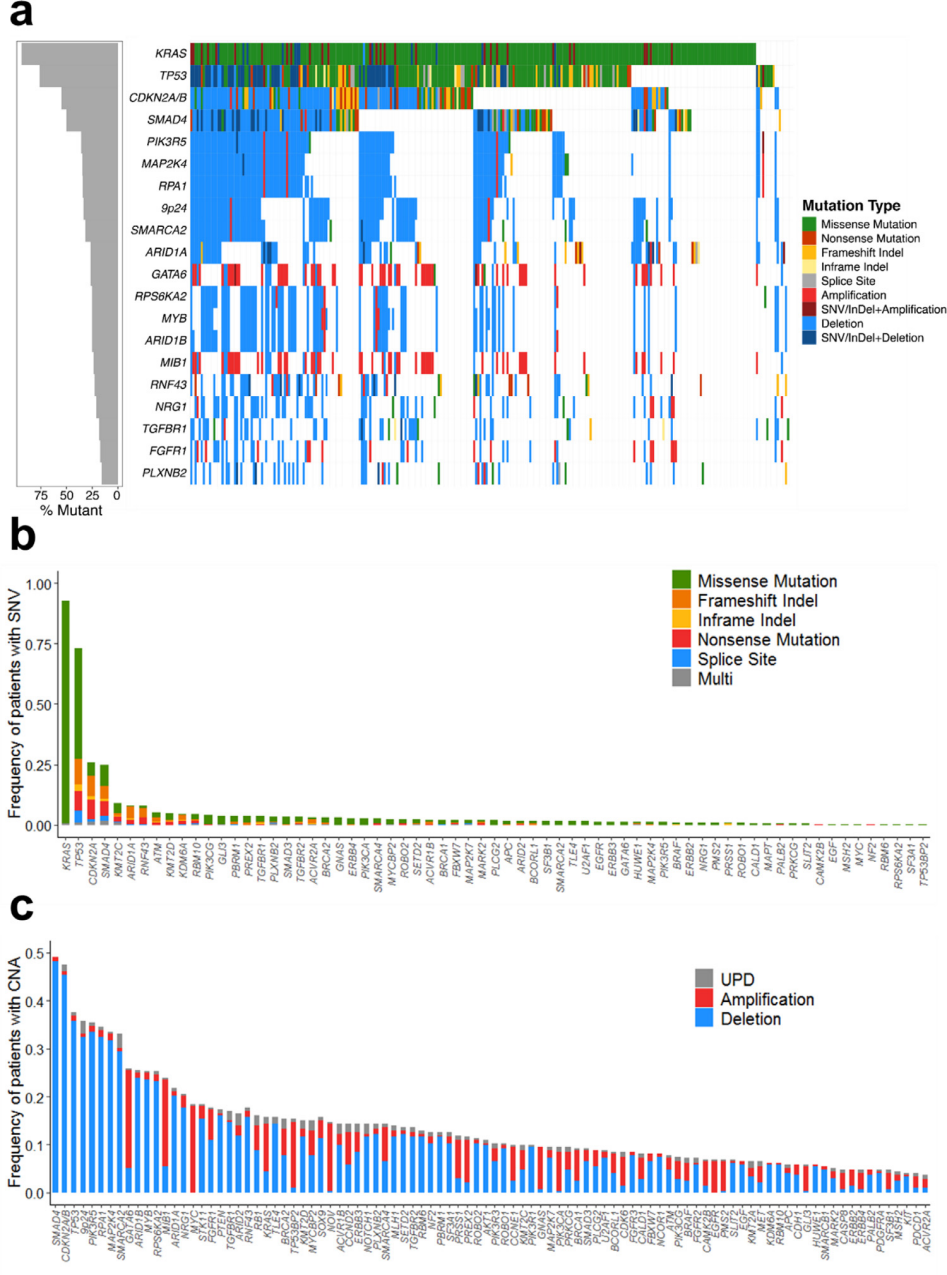
Overview of genetic alterations in R0-resected PDAC patients. (a) Landscape plot of the 20 most frequently altered genes in 293 PDAC patients. (b) Frequency and type of all SNVs within the 67 genes investigated for mutations and small insertions/ deletions (InDel). (c) Frequency and type of all CNAs within the 100 genes investigated for copy number alterations.

**Fig. 2:**
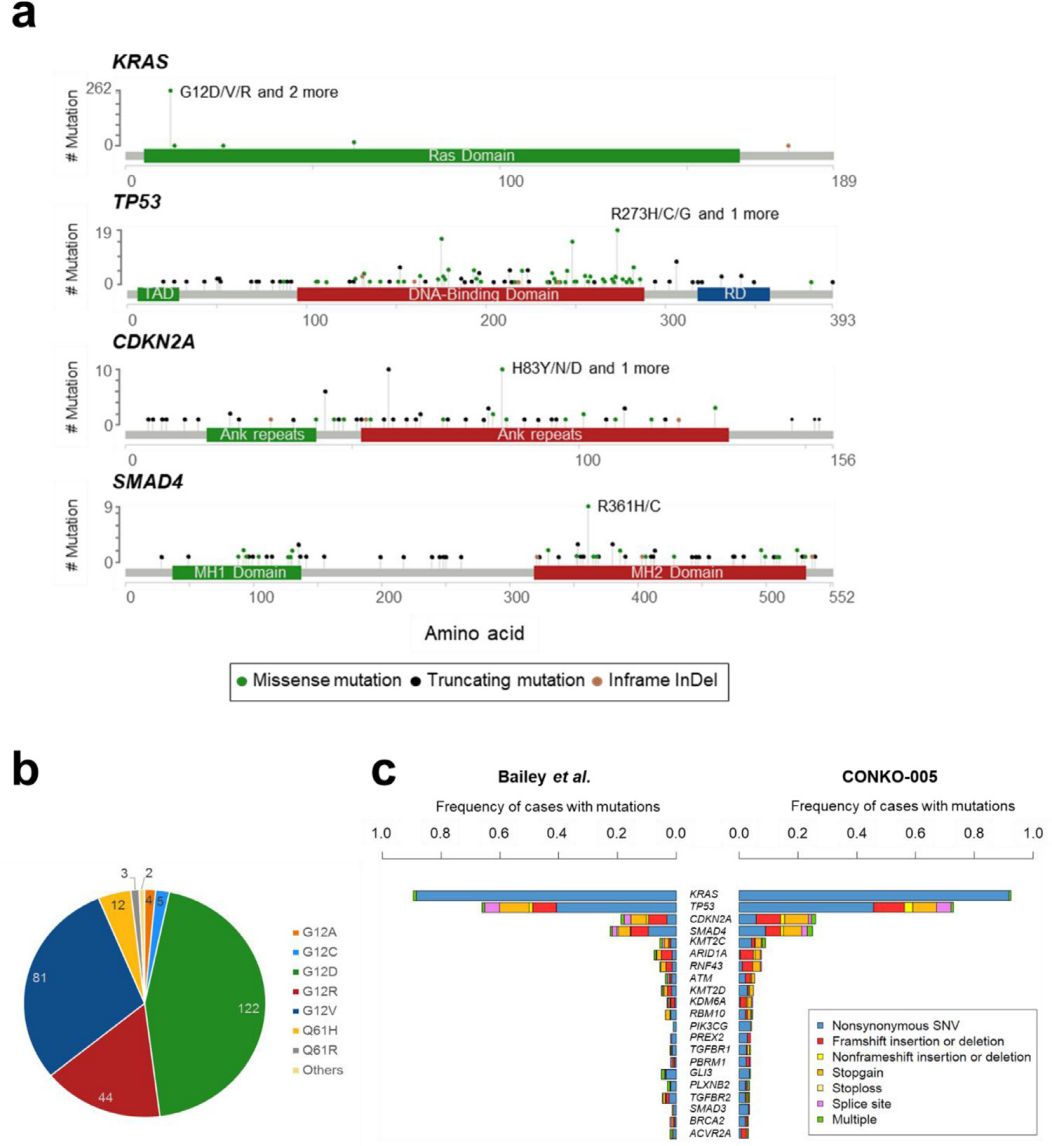
Detailed SNV profile of R0-resected PDAC patients. (a) Lollipop plot of four most commonly mutated genes. The most common SNVs are annotated for each gene. Modified from cbioportal [38,39]. (b) Overview of the different *KRAS* mutations with respect to their affected codon position and resulting amino acid change. The number of mutations for each variant is annotated. (c) Comparison of mutation frequencies and type with findings from Bailey et al. [6]. The 20 most frequently altered genes are shown in order of mutation frequency, based on the CONKO-005 cohort results. Mutations types are indicated by color. Abbreviations: TAD = trans-activation domain, RD = regulatory domain.

A total of 1086 SNVs and 4157 CNAs were identified in 293 patients (Fig. 1, Supplemental Tables S5 and S6). In 99% of all patients (*n* = 290), at least one genetic alteration was found, with a median of 4 (range: 0–23) SNVs and 11 (range: 0–66) CNAs per patient (Supplemental Figs. S4 and S5). Within the 67 genes analysed for SNVs, 58 were found to be recurrently mutated. The four most commonly mutated genes were *KRAS* (93%), *TP53* (74%), *CDKN2A* (27%), and *SMAD4* (27%), with mutations targeting known hot spots (e.g. *KRAS* G12) (Fig. 2a and b). Mutation types as well as mutation frequencies were comparable to previous PDAC sequencing studies (Fig. 2c) [5,6].

The major CNA loci were identified in genomic regions encoding for *SMAD4* (49%), *CDKN2A* (47%), and *TP53* (38%) as well as the 9p24 locus, containing the immune checkpoint regulators *CD274, PDCD1LG2,* and *JAK2* (35%). About 70% of all CNAs were deletions, nevertheless several well-known proto-oncogenes were found to be almost exclusively amplified (*KRAS, GATA6, MYC*). Gene specific focal CNAs, such as deletion of 9p21 with *CDKN2A/B* and gain of 18q11 with *GATA6*, as well as recurrent CNAs of entire chromosomes / chromosome arms, like loss of chromosome 6 and gain of 1q, were identified (Supplemental Fig. S6). The main pathways affected by SNVs and CNAs were the MAPK-pathway (99%), cell cycle control (92%) and TGFβ signalling (77%). Genes encoding for members of the PI3K/AKT pathway (65%) and genes involved in chromatin remodelling (71%) were also frequently affected in this cohort (Supplemental Fig. S7, Supplemental Table S7). The *EGFR* gene was found to be mutated in four patients (1.4%) and amplified in 19 patients (6.5%).

**Fig. 3:**
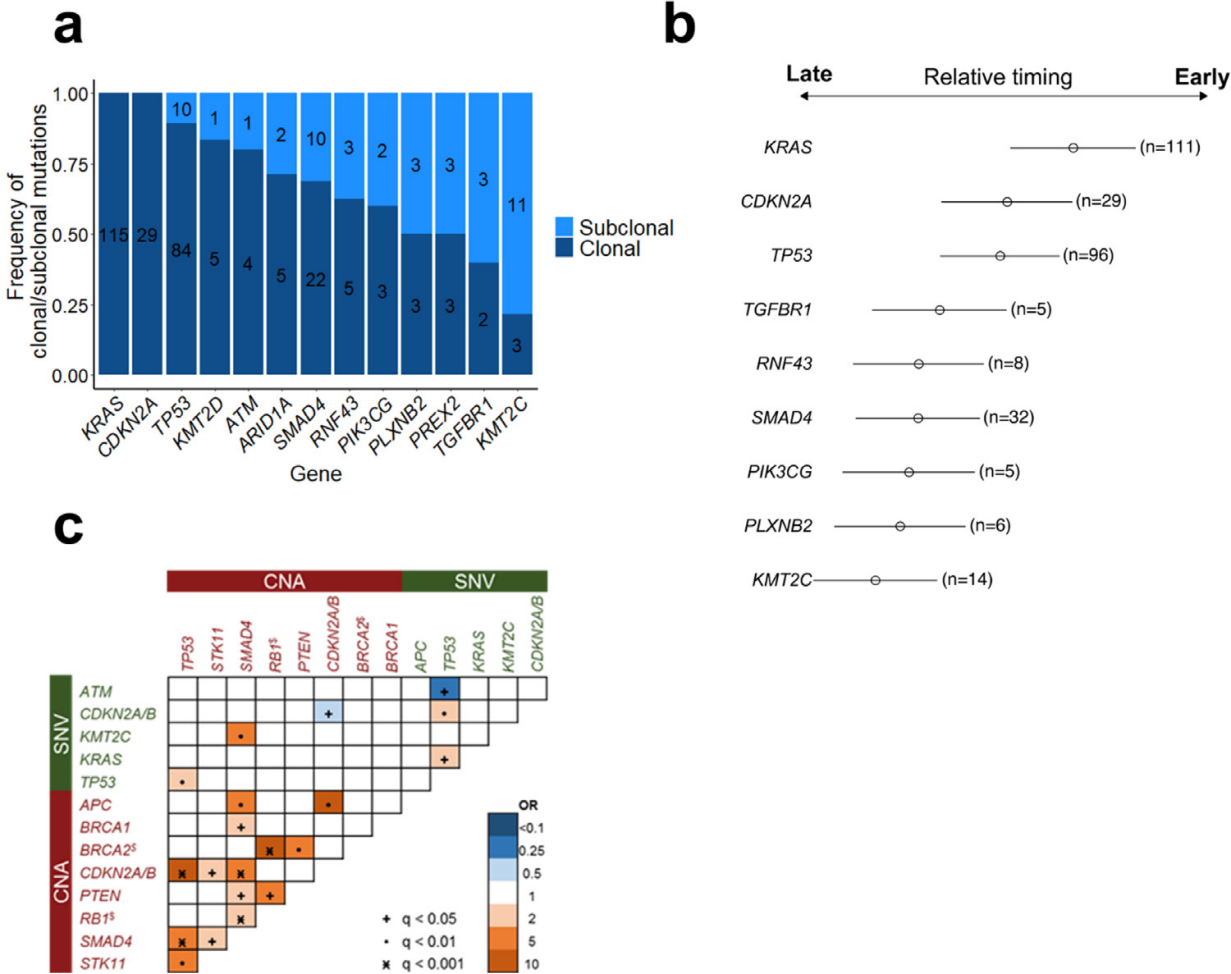
Overview of clonal hierarchies and genetic interaction pattern. (a) Clonality status of genes mutated in 124 patients without CNA in *KRAS*. (b) Bradley-Terry Model of all genes with sufficient mutational overlap with concomitant genetic events for model construction. 124 copy number neutral *KRAS* mutated patients with 2 or more additional mutations were used to calculate CNA adjusted VAFs. (c) Co-mutations and mutual exclusivity plot for all 293 patients. Genes mutated in at least 4% of patients and CNAs in all MLPA genes were included in the analysis. Significance levels of *q*-values (multiple testing corrected) are shown with symbols, odds ratio with colors (blue show different levels of mutual exclusivity, orange show different levels of co-mutation). $ = genes are located within same chromosomal region on chromosome 13q14.

To study preferred trajectories of disease evolution, we used variant allele frequencies (VAFs) to estimate the proportion of tumour cells carrying a given mutation and to identify clonal (in all cells) or subclonal mutations (in a fraction of cells). We applied this approach to 124 copy number neutral *KRAS* mutated patients with 2 or more oncogenic mutations from which we were able to calculate CNA adjusted VAFs to infer clonal architecture [17]. This approach identified genetic alterations in *KRAS, CDKN2A,* and *TP53* to be mostly clonal and thus likely to represent disease-initiating events (89 – 100% clonal), while mutations in *SMAD4* were more often subclonal. Mutations affecting *TGFBR1* and *KMT2C* (Fig. 3a and b) were likely to be later evolutionary events.

Next, we searched for pairwise gene associations, recognizing that pairs of genes could show a tendency to either co-occurrence or mutually exclusivity. In addition, we were interested to dissect the interplay of SNV and CNA acquisition. A total of 14 pairs were significantly associated with a false discovery rate <5%. The three major CNAs affecting the genes *SMAD4, CDKN2A,* and *TP53* showed a strong co-occurrence, which means that many patients have two or more of these genes concomitantly deleted. In addition, *TP53* mutations were often accompanied by mutations in *SMAD4, KRAS*, and *CDKN2A/B*. While mutually exclusive gene pairs often imply functional redundancy, such a pattern was rarely observed. Only *ATM* mutations hardly co-occurred with *TP53* mutations, especially with *TP53* activating mutations (Figs. 3c, S8, Supplemental Table S8).

### 3.2 Non-negative matrix factorization (NMF) reveals molecular subgroups with distinct clinical outcomes

**Fig. 4:**
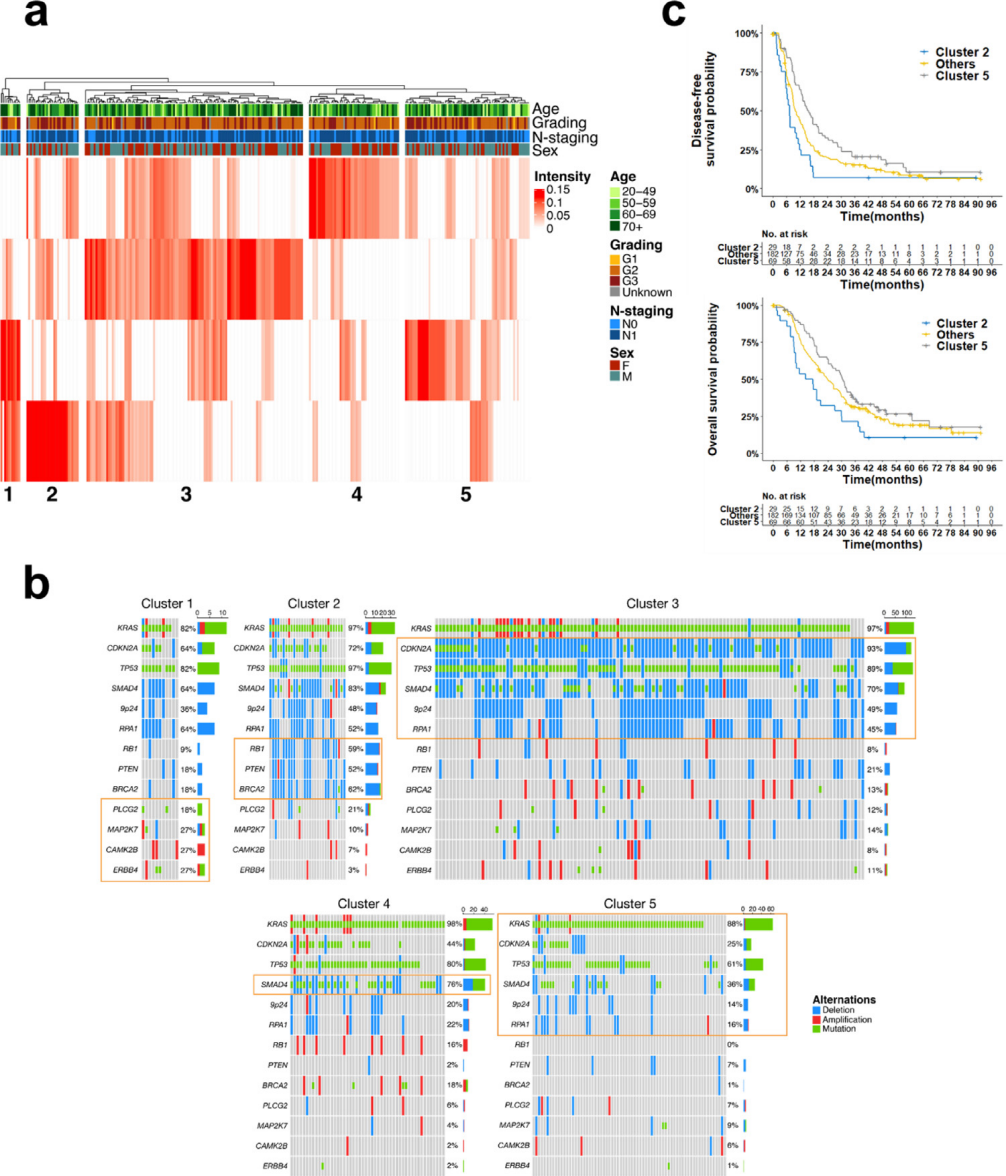
Unsupervised patient clustering using non-negative matrix factorization. (a) Heat map showing to which extent the four signatures extracted from 78 genetic alterations with NMF match for each patient of the cohort (intensity) (*n* = 283). Hierarchical clustering was used to group the patients into five distinct clusters. Additional annotations show their respective clinical baseline characteristics. (b) Exemplary genes showing the molecular biological differences between clusters. Significantly enriched/depleted genes are circled in orange. (c) Kaplan-Meier curves depicting DFS and OS from patients of clusters 2 and 5 compared to all other patients.

The complex genetic landscape in PDAC prompted additional analyses to better capture multiparametric patterns of mutational co-occurrence. NMF was performed to extract signatures in 78 genetic alterations (67 SNVs and 11 CNAs). The four extracted signatures were used to group the patients with hierarchical clustering into five robust patient clusters (Fig. 4a). To define the robustness of patient clusters, the stability of extracted signatures was tested with random step-wise subsampling of the patient cohort (Supplemental Fig. S3). While baseline characteristics such as age, gender, *N*-stage, and grading were distributed similarly between all clusters (Fig. 4a, Supplemental Table S9), a wide range of distinct genetic and clinical features could be attributed to each cluster (Supplemental Table S10). For example, tumours from patients in cluster 1 (*n* = 11) had significantly more mutations (8.6 vs 3.5 in all other groups) and a significant enrichment of mutations and amplifications in genes from the ERBB signalling pathway (*PLCG2, MAP2K7, ERBB4,* and *CAMK2B)*. Cluster 2 (*n* = 29) was enriched for copy-number deletions in well-known tumour suppressor genes (*RB1, BRCA2,* and *PTEN*), while cluster 3 (*n* = 121) contained numerous deletions affecting major PDAC gene loci (e.g. *CDKN2A/B, TP53*, and 9p24). In contrast, clusters 4 (*n* = 50) and 5 (*n* = 69) both had much fewer alterations per tumour (12.2 and 9.2 vs 25.9, 24.4, and 22.4 in clusters 1, 2, and 3). In cluster 4, this was mainly based on the absence of *CDKN2A/B* deletions, whereas in cluster 5 frequently altered genes, such as *SMAD4, CDKN2A/B, TP53,* and *KRAS,* were all less often affected. Of note, cluster 4 had a significant enrichment of *SMAD4* mutations and showed an overrepresentation of the transition base change C>A/G>T (*p* = 0.002) (Figs. 4b, S9).

Additionally, clusters 2 and 5 showed specific mRNA gene expression patterns. Cluster 2 associated significantly with overexpression of genes encoding receptors and effectors of the PI3K/AKT pathway (*MYB, MDM2*). In cluster 5, we saw overexpression of MAPK pathway activating genes (*RASGRP1, PDGFA,* and *PRKACB*) as well as the PI3K-AKT inhibitor *PTEN*, while several cell cycle control genes were decreased (*PKMYT1, SFN, CHEK2,* and *SKP2*) (Supplemental Fig. S10).

Notably, these genetic and transcriptomic differences were associated with clinical outcome. Patients in cluster 2 had significantly shorter DFS and OS times in multivariable cox regression analyses (HR=1.96, *p* = 0.002, and HR=2.06, *p* = 0.001). In contrast, cluster 5 patients showed longer DFS and OS (HR= 0.6, *p* = 0.002 and HR= 0.65, *p* = 0.015) (Figs. 4c, S11).

Furthermore, we could identify novel potential points of action for targeted therapies in respective clusters: in cluster 1 the prevalent mutations in *ERBB4, GNAS,* and *KMT2D* have been described as potential biomarkers [20], [21], [22], e.g. loss-of-function mutations in *KMT2D* were associated with sensitivity to BET inhibition in PDAC cells [23]. In cluster 2 with deletions in *RB1, BRCA2* and *PTEN*, for example, the oral pan-AKT Inhibitor MK-2206 has been shown to decrease tumour size and CA19–9 levels in PDAC patients with *PTEN* loss [24] and the PARP inhibitor olaparib prolonged progression-free survival in metastatic PDAC patients with *BRCA1/2* mutations [25]. Interestingly, patients in cluster 4 displayed a trend towards longer DFS and OS, when treated with erlotinib in combination with gemcitabine (GemErlo) as compared to the gemcitabine only treatment arm (DFS: *p* = 0.098; OS: *p* = 0.089) (Supplemental Fig. S12).

The largest cluster 3 showed no unique biological and/or clinical associations, likely due to persisting heterogeneity within this cluster requiring further subgrouping based on even larger patient cohorts. Nevertheless, our clustering based on the signatures extracted with NMF identified two well-defined patient clusters (clusters 2 and 5) with distinctive clinical outcome and potentially actionable genetic lesions as well one cluster with a trend for increased erlotinib sensitivity (cluster 4).

### 3.3 Increased responsiveness to erlotinib counteracts negative prognostic effect of *SMAD4* alterations especially in patients with low *MAPK9* expression

**Fig. 5:**
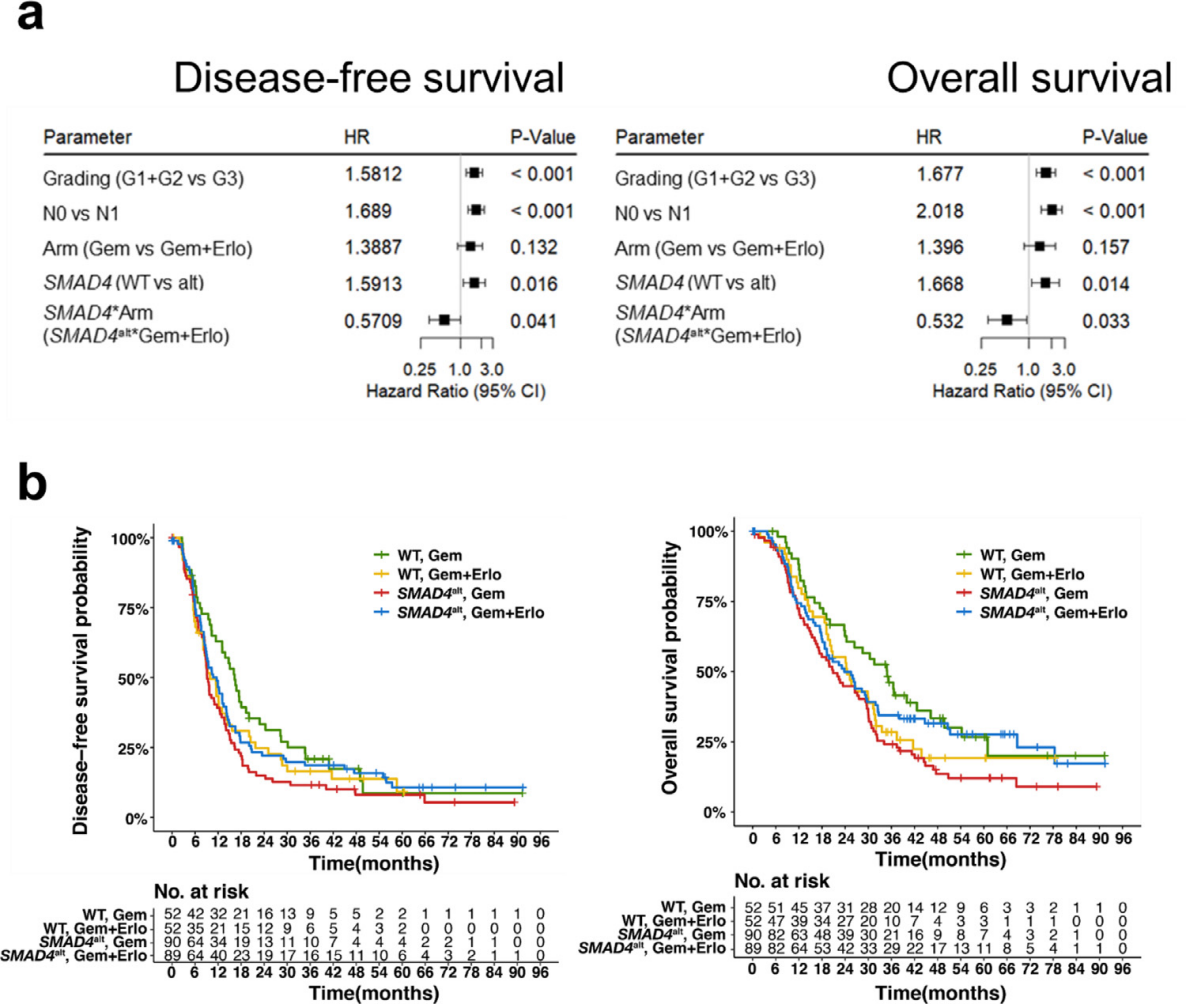
Erlotinib sensitivity in *SMAD4* altered PDAC patients. (a) Forrest plot of multivariable cox hazard model for the interaction of *SMAD4* status and treatment arm, containing all clinical baseline characteristics. (b) Kaplan-Meier curve comparing OS between both treatment arms in *SMAD4* altered (*SMAD4*^alt^) and *SMAD4* wild-type (WT) patients (2-sided log-rank test).

As previously shown for advanced PDAC [26], no predictive or prognostic effect on DFS or OS was observed with respect to the *EGFR* mutation or CNA status in this cohort (Supplemental Figure S13). The longer OS in the erlotinib treatment arm of cluster 4, which is enriched for *SMAD4* mutations, provided a first hint for a genetic alteration to be predictive for erlotinib sensitivity. Since most *SMAD4* SNVs were truncating mutations and almost all *SMAD4* CNAs were deletions suggesting similar loss of function consequences, we grouped patients harbouring at least one *SMAD4* alteration in a *SMAD4* altered subgroup (*SMAD4*^alt^; *n* = 179). While both *SMAD4^alt^* status and treatment arm alone were not prognostic for OS in a multivariable cox hazard analysis, the interaction test of both parameters correlated with a significant longer OS and DFS (HR= 0.53, *p* = 0.033 and HR= 0.57, *p* = 0.041, respectively) (Figs. 5a, S14). After correcting for the effect of the treatment arms, *SMAD4* alteration status itself became a negative prognostic marker for OS and DFS (OS: HR=1.67, *p* = 0.014; DFS: HR=1.59, *p* = 0.016, respectively) (Fig. 5b). In the gemcitabine treatment arm (Gem), *SMAD4*^alt^ patients had a significantly shorter DFS and OS compared to *SMAD4* wild-type (*SMAD4*^WT^) patients (log rank: DFS: *p* = 0.018, OS: *p* = 0.0078). At the same time, *SMAD4*^alt^ patients showed a trend towards longer OS when treated with GemErlo compared to gemcitabine alone without reaching significance (log rank: DFS: *p* = 0.209, OS: *p* = 0.056).

**Fig. 6:**
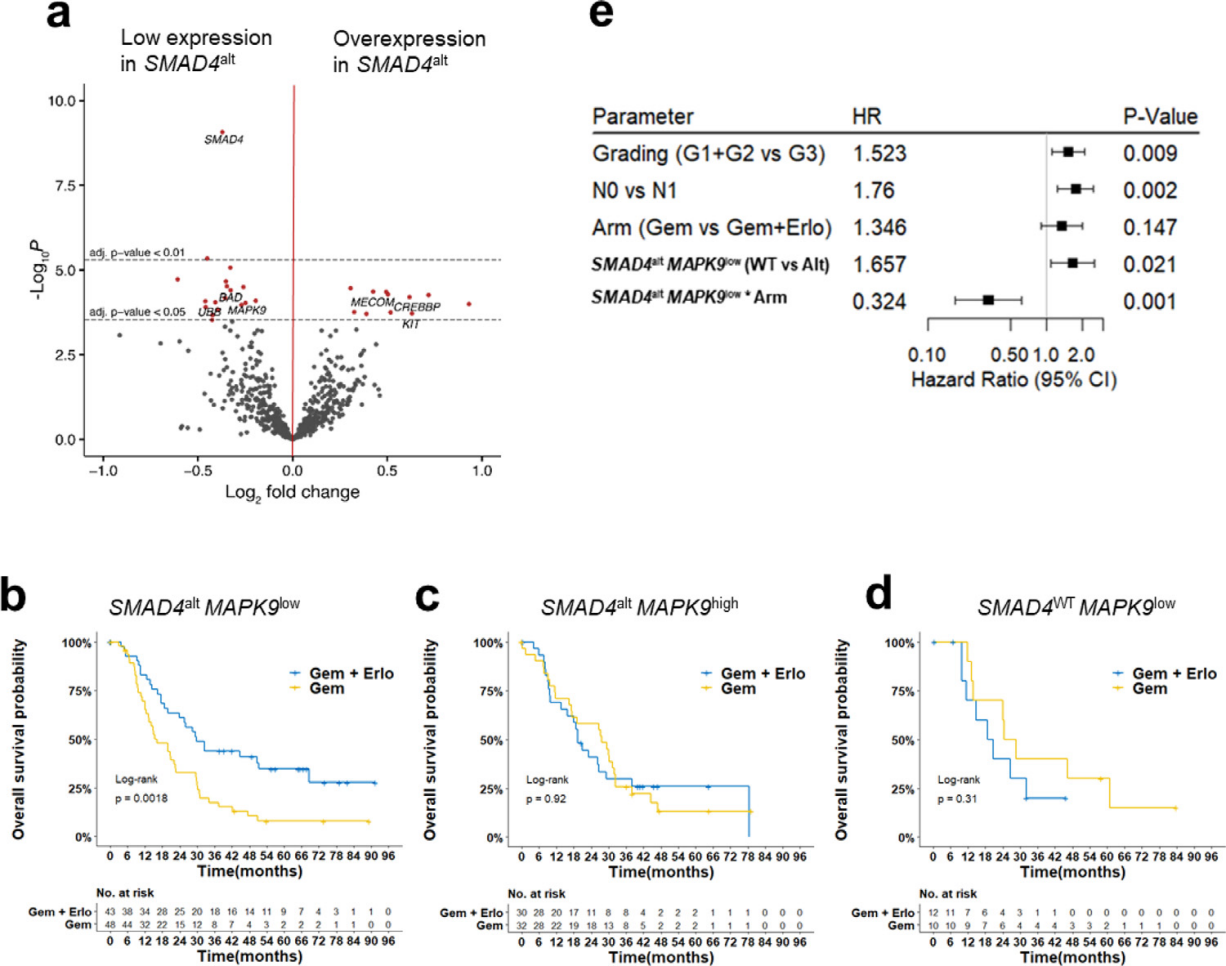
Erlotinib sensitivity in *SMAD4* altered PDAC patients is mediated by *MAPK9* expression levels. (a) Volcano plot of differential expression analysis between *SMAD4*^WT^ (baseline) patients and *SMAD4*^alt^ patients. Horizontal lines show significance levels of *p*-values (multiple testing adjusted with Benjamini-Hochberg). The 50 variants with the lowest *p*-value are labelled. Kaplan-Meier curves comparing OS between both treatment arms in patents according to their (b) *SMAD4*^alt^*MAPK9*^low^, (c) *SMAD4*^alt^*MAPK9*^low^, (d) *SMAD4^WT^MAPK9*^low^ status (2-sided log-rank test). (e) Forrest plot of multivariablecox hazard model for OS showing the interaction of *SMAD4*^alt^*MAPK9*^low^ status and treatment arm, containing all clinical baseline characteristics.

**Fig. 7:**
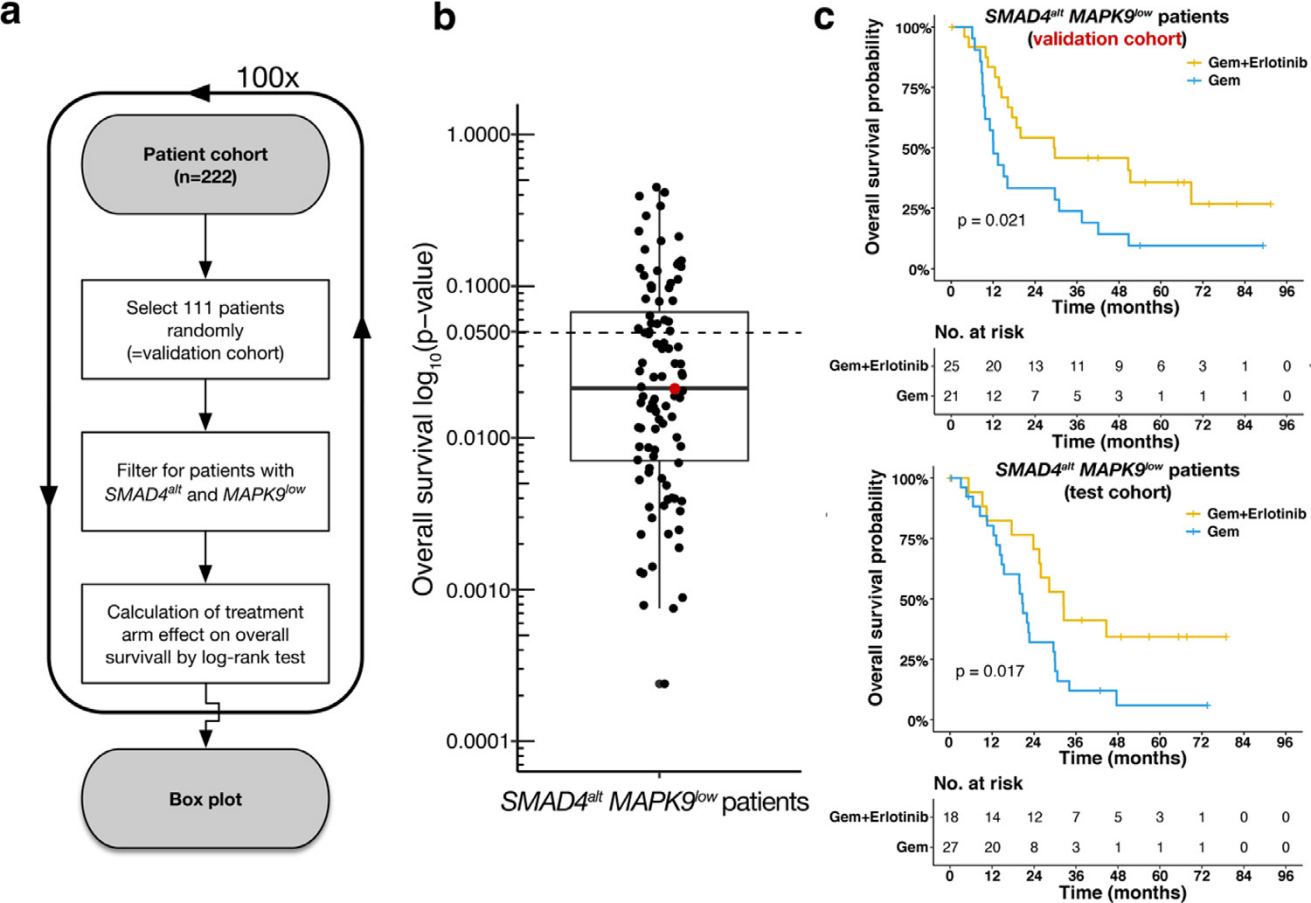
Validation of additive erlotinib in patients with a *SMAD4*^alt^*MAPK9*^low^ status. (a) A validation cohort and respectively a test cohort consisting of 111 patients each were picked randomly from the patient cohort (*n* = 222). This step was repeated 100 times. (b) Each dot represents one out of 100 randomly assigned validation cohort. The box plot (median: 0.021, upper quartile: 0.068, lower quartile: 0.007) was generated based on overall survival differences according to the two treatment arms (i.e., Gem + Erlotinib and Gem) in patients with a *SMAD4*^alt^*MAPK9*^low^ status. *P*-values derive from log-rank tests for all 100 randomly selected validation cohorts. (**c)** The validation cohort with a *p*-value closest to the median of all *p*-values, as visualised by the red dot, is shown exemplary. Kaplan-Meier curves comparing OS between both treatment arms for validation and test cohorts are shown for these specific validation and test cohorts.

In order to get insights into differentially activated pathways with respect to the underlying *SMAD4^alt^* status, we compared gene expression profiles from 153 *SMAD4*^alt^ and 69 *SMAD4*^WT^ PDAC patients. After correcting for multiple testing, a total of 11 and 19 genes were significantly up- or down-regulated in *SMAD4*^alt^ patients, respectively (Fig. 6a, Supplemental Table S11). A careful literature search for the relation with carcinogenesis and/or EGFR pathway inhibition of these 30 differentially expressed genes, pointed us to the down-regulated Jun-kinase *MAPK9,* a known transcriptional activator of the JNK pathway. As increased JNK activation has been shown to mediate acquired resistance to EGFR inhibition by bypass signalling [27], we hypothesized that effectiveness of EGFR inhibition might be altered according *MAPK9* expression levels in *SMAD4^alt^* patients*.* Integration of *SMAD4* genetic aberration status with *MAPK9* gene expression levels grouped *SMAD4*^alt^ patients into patients with low (91/222= 40.9%) or high (62/222=27.9%) *MAPK9* expression (Supplemental Table S12). Strikingly, the beneficial effect of erlotinib was restricted to *SMAD4*^alt^ patients with low *MAPK9* expression (DFS: HR=0.49; test for interaction, *p* = 0.02) and OS (HR= 0.32; test for interaction, *p* = 0.001). No differences were observed neither in *SMAD4*^alt^ patients with high *MAPK9* expression nor in *SMAD4*^WT^ patients with low or high *MAPK9* expression levels (Fig. 6c and d). To further validate this finding, we randomly divided our cohort in a test and a validation cohort of equal size (*n* = 111, respectively). Next, we compared OS of *SMAD4*^alt^ *MAPK9*^low^ patients within the test cohort according to both treatment arms (Fig. 7a). After 100 random assignments a median *p*-value of 0.021 was observed, confirming the beneficial effect of additive erlotinib over gemcitabine alone in this specific genotype (upper quartile: 0.068, lower quartile: 0.007) (Fig. 7b). For the randomly selected cohorts with a *p*-value closest to the median, both the test and the validation cohort resulted in a significantly increased OS in *SMAD4*^alt^ *MAPK9*^low^ patients treated with additive erlotinib (Fig. 7c).

In summary, *SMAD4*^alt^ patients had a significantly shorter OS and DFS when treated with gemcitabine alone, an effect that was negated when erlotinib was added to the adjuvant chemotherapy regimen, especially in *SMAD4*^alt^ patients with low *MAPK9* expression levels.

## 4 Discussion

Several seminal studies have defined the genetic landscape of PDAC over the last decade [4], [5], [6], and paved the ground for our panel design. As molecular data obtained from prospective and randomised clinical trials are rare to date [28], we speculated that signatures extracted from comprehensive SNV and CNA data through NMF factorization might identify patient subgroups with different response to gemcitabine ± erlotinib. We defined five tumour subgroups with important differences with respect to affected gene frequencies, underlying base change signatures, associated deregulated gene expression profiles and different survival outcome. Tumours from patients in cluster 5 had only few alterations in addition to the initiating *KRAS* mutations with increased MAPK pathway expression as well as high levels of *PTEN*. This implies that these patients’ tumours were driven by early PDAC events of hyperactive *KRAS*. In contrast, cluster 2 showed an enrichment of deletions in three major tumour suppressors (*PTEN, RB1*, and *BRAC2*). This lead to an uncontrolled activation of the PI3K-/AKT signalling pathway. Therefore, tumours in cluster 2 did not seem to solely rely on the initial MAPK pathway deregulation but have evolved to deregulate a second cell proliferation pathway with the effect of a more aggressive tumour biology leading to decreased DFS and OS.

Although these findings cannot directly be translated into clinical practice, they open new avenues for combined treatment approaches that should target different deregulated pathways based on respective patient clusters at the same time. This approach also helped to identify a genetically defined subgroup of PDAC patients that showed longer survival when treated with GemErlo in this phase 3 trial. Since Moore et al. showed some improvement of OS and DFS in erlotinib treated patients with advanced PDAC [29], several predictive biomarkers have been proposed. For example, *KRAS* mutation status was shown to be associated with improved OS after erlotinib treatment in advanced PDAC in smaller patient series [26,30]. In preclinical models, cell lines with the so-called “classical” Collison subtype, defined by *GATA6* overexpression, were shown to exhibit increased erlotinib sensitivity [31]. In our study, neither *KRAS* mutations status nor *GATA6* amplifications were associated with erlotinib sensitivity, which might be due to the fact that our patients were treated at an earlier disease stage. However, we found *SMAD4* alterations to be significantly associated with OS and DFS in a treatment dependant manner. While *SMAD4* is widely accepted as a poor prognostic biomarker [9], it has not been postulated in the context of predicting response to EGFR inhibition. Erlotinib has been shown to have similar or even higher affinities to other protein kinases than EGFR [32]. As neither *EGFR* expression levels nor genetic *EGFR* alterations were predictors of response in our study, contribution of erlotinib off-targets are conceivable. In line with this hypothesis, *SMAD4*^alt^ tumours showed downregulation of the Jun-kinase *MAPK9*. Increased JNK activation has been shown to mediate acquired resistance to EGFR inhibition by bypass signalling [27]. In fact, we identified *SMAD4* alterations with decreased *MAPK9* expression, identified in 91 patients of this study cohort, as a predictive biomarker for erlotinib response in R0-resectable PDAC patients. Since the CONKO-005 study was the first major clinical trial comparing erlotinib treatment with standard of care in R0-resected patients, our cohort is unique in its size and homogeneity. While this gave us the opportunity to analyse previously overseen genetic interactions, it made it impossible to validate our findings in a suitable second patient cohort. Even though we performed extensive self-validation to further strengthen the reliability of our analysis, the missing independent validation is a limitation of our study. Of note, a recent study showed a previously unappreciated function for tumour cell-intrinsic SMAD4 by promoting anti-tumour immunity in PDAC. This function could be attributed to a direct link between SMAD4 and EGFR through a transcriptional axis involving KDM3A, KLF5, and SMAD4 converging on EGFR [33]. As erlotinib sensitized tumors to combination immunotherapy *in vivo* [33], it will be of interest to study the combination of EGFR inhibition with immunotherapies in PDAC patients with *SMAD*4 aberrations. Another promising combinatorial approach was recently shown using simultaneous EGFR/c-RAF inhibition [34], further indicating that the full therapeutic potential of erlotinib in PDAC is yet to be defined.

One might question the role of Gemcitabine in curable PDAC as mFOLFIRNOX showed superiority in terms of longer DFS and OS as compared to gemcitabine alone in R0-resected PDAC patients recently [35]. However, real-world data about the adjuvant use of mFOLFIRINOX are not yet available. Median age in PDAC patients is 70 to 75 years and elderly patients are often not eligible for an intensified adjuvant chemotherapy. For these patients, Gemcitabine remains a well-documented and recommended option in the postoperative situation [36]. While a transcriptomic signature has recently been identified to predict Gemcitabine sensitivity [37], our data extend current precision medicine approaches with respect to the use of EGFR inhibitors in PDAC. Further analysis are now warranted to dissect the precise mechanisms of EGFR inhibition in PDAC.

## Funding

Else Kröner-Fresenius Foundation, Wegener Foundation, German Cancer Aid.

## Declaration of Competing Interest

Dr. Damm reports personal fees from Roche, Novartis, AbbVie, Astra Zeneca outside the submitted work. Dr. Sinn reports personal fees from Sanofi, Astra Zeneca, Amgen, Servier, MSD, Incyte, Pfizer outside the submitted work. Dr. Bullinger reports personal fees from Abbvie, Amgen, Astellas, Bristol-Myers Squibb, Celgene, Daiichi Sankyo, Gilead, Hexal, Janssen, Jazz Pharmaceuticals, Menarini, Novartis, Pfizer, Sanofi, and Seattle Genetics outside the submitted work. Dr. Keilholz reports personal fees from Bristol-Myers Squibb, MSD, Merck Serono, Pfizer, Astra Zeneca outside the submitted work. All other authors have declared no conflicts of interest.

## Appendix Supplementary materials

Image, application 1

Image, application 2

Image, application 3
