# Supplementary material for: A genetically defined signature of responsiveness to erlotinib in early-stage pancreatic cancer patients: Results from the CONKO-005 trial
Source: eBioMedicine. 2021 Apr 13;66:103327. doi: 10.1016/j.ebiom.2021.103327 (PMC8054140; doi:10.1016/j.ebiom.2021.103327)
Supplement: Supplementary file 1 [file mmc1.docx]

**Caption for supplementary material** **(EBIOM-D-21-00032)**

1. Supplementary file “20210315_Hoyer et al_Supplement_revised”:

caption: Supplementary Methods, Figures, and Tables

1. Supplementary file “20200829_Hoyer_et_al_Supplemental_Tables”:

caption: Supplementary Tables S1, S5, S6, S8, S11
