# Supplementary material for: A genetically defined signature of responsiveness to erlotinib in early-stage pancreatic cancer patients: Results from the CONKO-005 trial
Source: eBioMedicine. 2021 Apr 13;66:103327. doi: 10.1016/j.ebiom.2021.103327 (PMC8054140; doi:10.1016/j.ebiom.2021.103327)
Supplement: Supplementary file 2 [file mmc2.pdf]

Supplemental Information

A genetically defined signature of responsiveness to erlotinib in early-stage pancreatic cancer patients: results from the CONKO-005 trial

Hoyer K[1], Hablesreiter R[1], Inoue Y[2], Yoshida K[2], Briest F[1], Christen F[1], Kakiuchi N[2], Yoshizato T[2], Shiozawa Y[2], Shiraishi Y[3], Striefler JK[1], Bischoff S[1], Lohneis P[4,5], Putter H[6], Blau O[1], Keilholz U[7], Bullinger L[1], Pelzer U[1], Hummel M[4], Riess H[1], Ogawa S[2,8,9*], Sinn M[1,10*], Damm F[1,11,12*]

1. Charité – Universitätsmedizin Berlin, corporate member of Freie Universität Berlin, Humboldt-Universität zu Berlin, and Berlin Institute of Health, Department of Hematology, Oncology, and Tumor Immunology, Berlin, Germany
2. Department of Pathology and Tumor Biology, Graduate School of Medicine, Kyoto University, Kyoto, Japan
3. Laboratory of DNA information Analysis, Human Genome Centre, Institute of Medical Science, The University of Tokyo, Tokyo, Japan
4. Charité – Universitätsmedizin Berlin, corporate member of Freie Universität Berlin, Humboldt-Universität zu Berlin, and Berlin Institute of Health, Institute of Pathology, Berlin, Germany
5. Institute of Pathology, University of Cologne, Cologne, Germany
6. Department of Biomedical Data Sciences, Leiden University Medical Center, Leiden, The Netherlands
7. Charité – Universitätsmedizin Berlin, corporate member of Freie Universität Berlin, Humboldt-Universität zu Berlin, and Berlin Institute of Health, Charité Comprehensive Cancer Center, Berlin, Germany
8. Institute for the Advanced Study of Human Biology (WPI-ASHBi), Kyoto University, Kyoto, Japan
9. Department of Medicine, Centre for Haematology and Regenerative Medicine, Karolinska Institute, Stockholm, Sweden
10. Department of Oncology, Hematology and Bone Marrow Transplantation with Division of Pneumology, University Medical Center Hamburg-Eppendorf, Hamburg, Germany
11. German Cancer Consortium (DKTK), partner site Berlin, Berlin, Germany
12. German Cancer Research Center (DKFZ), Heidelberg, Germany

* S.O., M.S., F.D. contributed equally as senior authors.

Supplemental Methods

Patients

All patients were enrolled in the CONKO-005 study, an open-label, multicenter, randomized phase III trial investigating the addition of erlotinib to gemcitabine compared to gemcitabine only as adjuvant therapy. [1] For detailed eligibility criteria see Sinn et al.[1] After R0 resection, 436 PDAC patients were enrolled and randomly assigned to either the experimental arm (GemErlo[n=219]) or the standard treatment arm (Gem[n=217]). Patients in the GemErlo arm were treated with Gemcitabine 1,000 mg/m2 days 1, 8, 15 every 4 weeks in combination with erlotinib 100 mg orally once per day on days 1 to 28 every 4 weeks, and those in the gemcitabine alone arm were treated with the same dose and interval of gemcitabine monotherapy, both for 24 weeks therapy. Formalin embedded resected pancreatic tissue was collected from 331 adult patients (age, 24-82 years). Written consent was obtained in accordance with the Declaration of Helsinki and with ethical approval obtained from the local ethics committee.

Sample preparation and quality control

Paraffin-embedded blocks from the preintervention surgical resection were assessed visually for determination of highest tumor content areas. Samples with a tumor content below 10% were excluded from this study. Samples with a tumor content below 50% were macrodissected in order to enrich the tumor cell fraction; those with a tumor content above 50% were directly processed. DNA and RNA were extracted using the half-automated Maxwell system (Promega) with the Maxwell 16 FFPE Tissue LEV DNA Purification Kit (Promega) and the RSC RNA FFPE Kit (Promega) according to the manufacturer's instruction. Sufficient amounts of DNA and RNA with adequate quality were extracted from 307 and 250 FFPE samples, with a tumor content of at least 10%, respectively (Supplemental Table S1). Additionally, DNA from 20 tonsillectomy FFPE tissue samples was extracted as non-paired normal tissue. After quality controls of raw sequencing results, 293 patients with a median age of 64 years were included in this study (Table 1).

Mutation analysis by targeted sequencing

307 patient samples as well as 20 non-paired normal samples were screened with a self-designed custom panel. The panel covered full-length coding regions of 67 genes described as "significantly mutated" in one of the two largest PDAC sequencing studies,[2,3] shown as clinically relevant in PDAC,[4-6] genes that were previously included in PDAC gene panels,[5,7] and/or representing major players in the EGFR pathway (Supplemental Table S2). The custom Agilent SureSelectXT Target Enrichment System for Illumina Paired-End Multiplexed

Sequencing was used according to the manufacturer's instructions with the following adaptation: for end repair and a-tailing, the KAPA Hyper Prep Kit (Kapa Biosystems) was used. Libraries were paired-end sequenced with a mean sequencing depth of ~600x and a minimal reading depth of 200x on a HiSeq 2000 sequencer (Illumina). Sequence alignment and mutation calling was performed using our in-house pipeline 'Genomon v.2.5.0', as previously described.[8] Reads that had either a mapping quality score of <25, a base quality score of <30, or 5 or more mismatched bases were excluded from the analysis. Candidate mutations with i) a variant allele frequency (VAF) ≥0.04; and ii) a EBcall[9] (Empirical Bayesian mutation calling) p-value $\leq 1\times10{-}10$ were adopted and filtered further. We excluded: i) synonymous mutations and variants without complete ORF information; ii) known variants listed in the 1000 Genomes Project (version May 2011), NCBI SNP database (dbSNP) build 131; National Heart, Lung, and Blood Institute (NHLBI) Exome Sequencing Project (ESP) 5400, the Human Genome Variation Database (HGVD; October 2013 release), or our in-house SNP database; iii) variants present only in unidirectional reads; iv) variants occurring in repetitive genomic regions; v) variants with <2 supporting reads; and vi) all variants found in non-paired normal samples (n=20) showing an allele frequency of >0.0025. Finally, mapping errors were removed by visual inspection in the Integrative Genomics Viewer (IGV) browser.[10]

To reduce the likelihood of false single nucleotide variant (SNV) calling, we established a validation pipeline using a combination of several variables such a SNV frequency, EBcall p-value, DNA quality, and sequencing duplication rate. A total of 219 potential SNVs representing 17% of all detected variants were investigated in a second independent experiment either by amplicon-based targeted deep sequencing (n=195) or ddPCR (n=24) as previously described.[11,12] With a mean coverage of 88102x, we could validate 210 variants, which led to a high validation rate of 96%.

Copy-number alterations (CNA) detection by targeted sequencing

Copy-number analysis was performed as previously reported using an in-house pipeline CNACS (https://github.com/papaemmelab/toil_cnacs) (Y. Shiozawa and S.Ogawa, manuscript in preparation),[13] in which the total number of reads covering each bait region and the allele frequency of heterozygous single-nucleotide polymorphisms (SNP) detected by targeted sequencing were used as input data. To this aim, 1305 probes, spread across the entire genome and serving as a chromosomal backbone, were included in the custom panel. Together with 400 gene specific probes (3-6 per gene), targeting 100 genes previously described as drivers in PDAC (Supplemental Table S3), it enabled CNA detection. For 171 patients we could detect CNAs based on the targeted sequencing data. In the remaining 112 samples, we generated information about local copy number changes in 11 genes based solely

on a Multiplex Ligation-dependent Probe Amplification (MLPA) assay. For 10 patients we were not able to obtain copy number data with either method (Figure S2).

Multiplex Ligation-dependent Probe Amplification (MLPA) assay

To validate potential CNAs identified by targeted sequencing and identify CNAs in samples not suitable for CNA detection by targeted sequencing, we used commercially available MLPA assays according to the manufacturer's instructions. Therefore, 70 ng of DNA were denatured, hybridized to probes of either the SALSA MLPA probe mix P294-C1 Tumor Loss or the P175-B1 Tumor Gain (MRC Holland), ligated, and amplified. Each probe resulted in an amplicon with a distinct length. Subsequently the amplicon mix was separated with capillary electrophoresis and the ratio of target probe to backbone probe was calculated. Probes with a ratio below 0.8 were considered as deleted and above 1.2 as amplified. Data analysis was performed using Coffalyser.Net (version 140721.1958).

Expression profiling with nCounter Technology

RNA concentration was measured on Quantus Fluorometer using the QuantiFluor RNA System (Promega) and RNA degradation levels were determined with the RNA 6000 Nano Kit on a 2100 Bioanalyzer (Agilent). The NanoString nCounter Flex system was used to run a customized version of the PanCancer Pathways Panel (770 genes representing 13 canonical pathways in cancer, 606 Pathway genes, 124 cancer driver genes and 40 reference genes panels) with 28 additional genes (Supplemental Table S4). Raw NanoString counts were normalized to internal positive control probes and housekeeping genes using nSolver Software (NanoString Technologies, WA, USA) version 4.0, according to default parameters, with background threshold count value set to 20. After quality controls, 230 samples were analyzed. Differential expression analysis and volcano plot generation was done using the nCounter Advanced Analysis Pugin (version 2.0.115). Genes were tested for differential expression in response to each selected covariate. For each gene, a single linear regression was fit using all selected covariates to predict expression and false discovery rate (FDR) was estimated according to the Benjamini-Hochberg procedure.[14]

Statistical analysis

Statistical analyses were performed using IBM SPSS statistics (version 24) and R (version 3.6.1). Co-occurrence and mutational exclusivity was calculated with Fisher's exact test and subsequently corrected for multiple hypothesis testing using the Benjamini–Hochberg method[14]. To model clonal composition we used a modified version of the SciClone Bioconductor package as previously described2.[15,16] Non-negative matrix factorization (NMF), which is an unsupervised machine-learning approach, was performed using the R-package

Bratwurst (version 1.0)[17] to extract signatures in SNV and CNA patterns of investigated patients. We used the dichotomized mutation status of all 67 panel genes as well as the dichotomized CNA status of the 11 genes included in the MLPA tumor loss kit as input. The optimal number of signatures (factorization rank k=4), was determined based on several quality criteria (*e.g.*, Frobenius reconstruction error, cophenetic correlation coefficient, and amari type distance). Step-wise subsampling of the patient cohort (minus five patients per step) was performed to ensure a high stability of the identified signatures and, therefore, a high cluster stability (Supplemental Figure S3). For alteration enrichment analysis in the identified patient clusters, we calculated odds ratios for each alteration and compared them with two-tailed t-tests. Variants were considered significantly enriched when, after multiple testing correction, q-value was <0.05. The Fisher's exact test was subsequently used to compare amount of alterations and base change pattern. Cox models were used for time to-event variables (OS and DFS), and p-values were calculated using the Wald test. Multivariate cox proportional hazards models were used to investigate variables associated with survival endpoints To select input for the multivariate cox proportional hazards models, univariate cox regression analysis of all clinical variables were carried out. Primary analysis endpoint was OS, followed by exploratory analysis into DFS. While genetic alterations were encoded as either "altered" or "normal", clinical variables were dichotomized as follows: tumor size (T1/2 vs T3/4), lymph node involvement (N0 vs N+), grade (grade 1 to 2 vs grade 3), Karnofsky performance status (90% to 100% vs < 90%), age (≤65 vs >65), sex (m vs f), treatment arm (Gemcitabine vs Gemcitabine+Erlotinib). Variables with an adjusted p-value ≤ 0.05 were included in subsequent multivariate analysis. Kaplan-Meier analysis was performed to construct survival curves and log-rank test was applied to evaluate differences between subgroups. The Hardy-Weinberg equilibrium was not considered.

Supplemental Figures

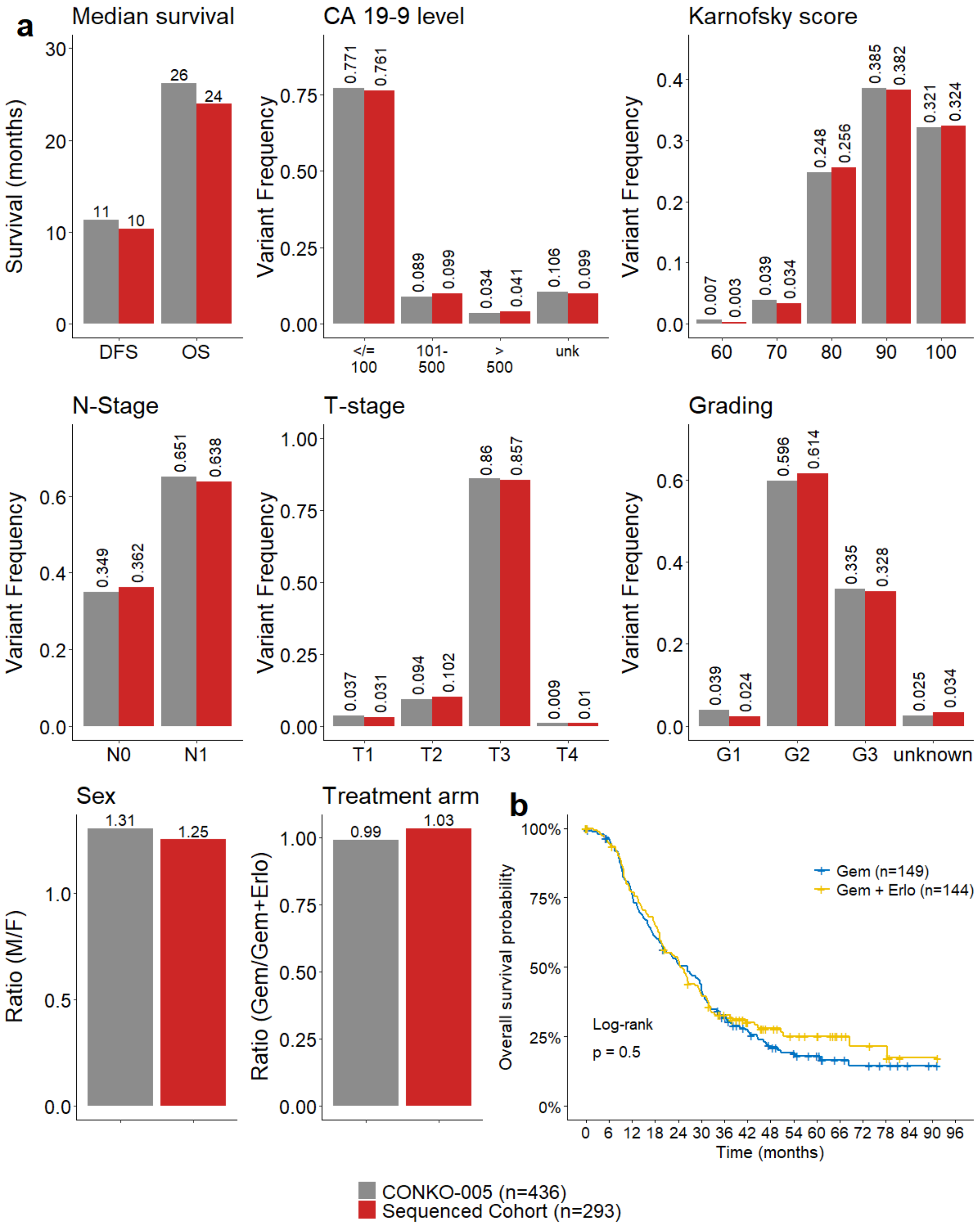

Figure S1: a) Comparison of baseline characteristics between the entire CONKO-005 study population (grey) and the subset of 293 patients sequenced within this study (red) indicating a high comparability of both patient groups. **b)** Overall survival of the 293 PDAC patients according to the two treatment arms of the CONKO-005 trial: Gemcitabine (blue) vs. Gemcitabine + Erlotinib (yellow).

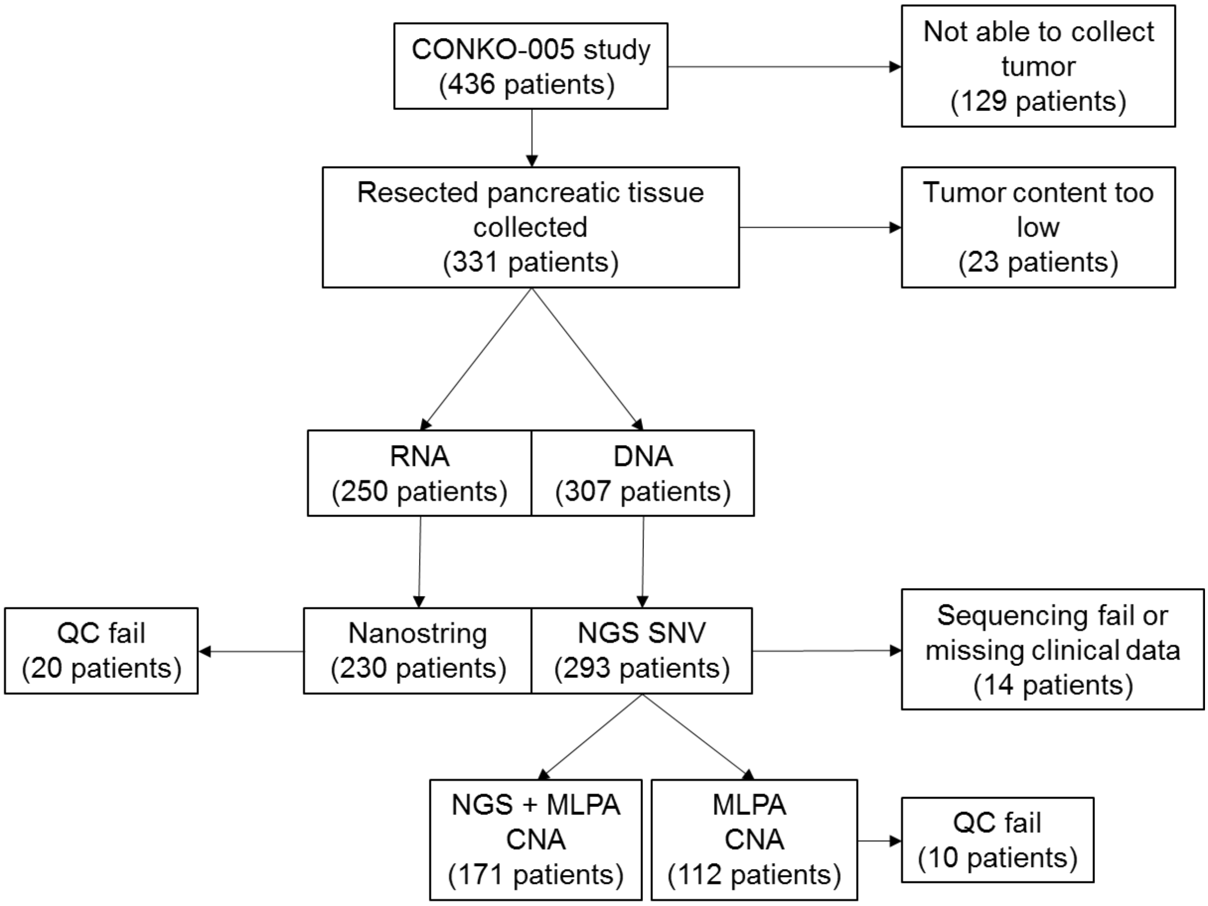

Figure S2: Flow chart of sample procession, showing the number of samples used for each analysis of the CONKO-005 trial cohort.

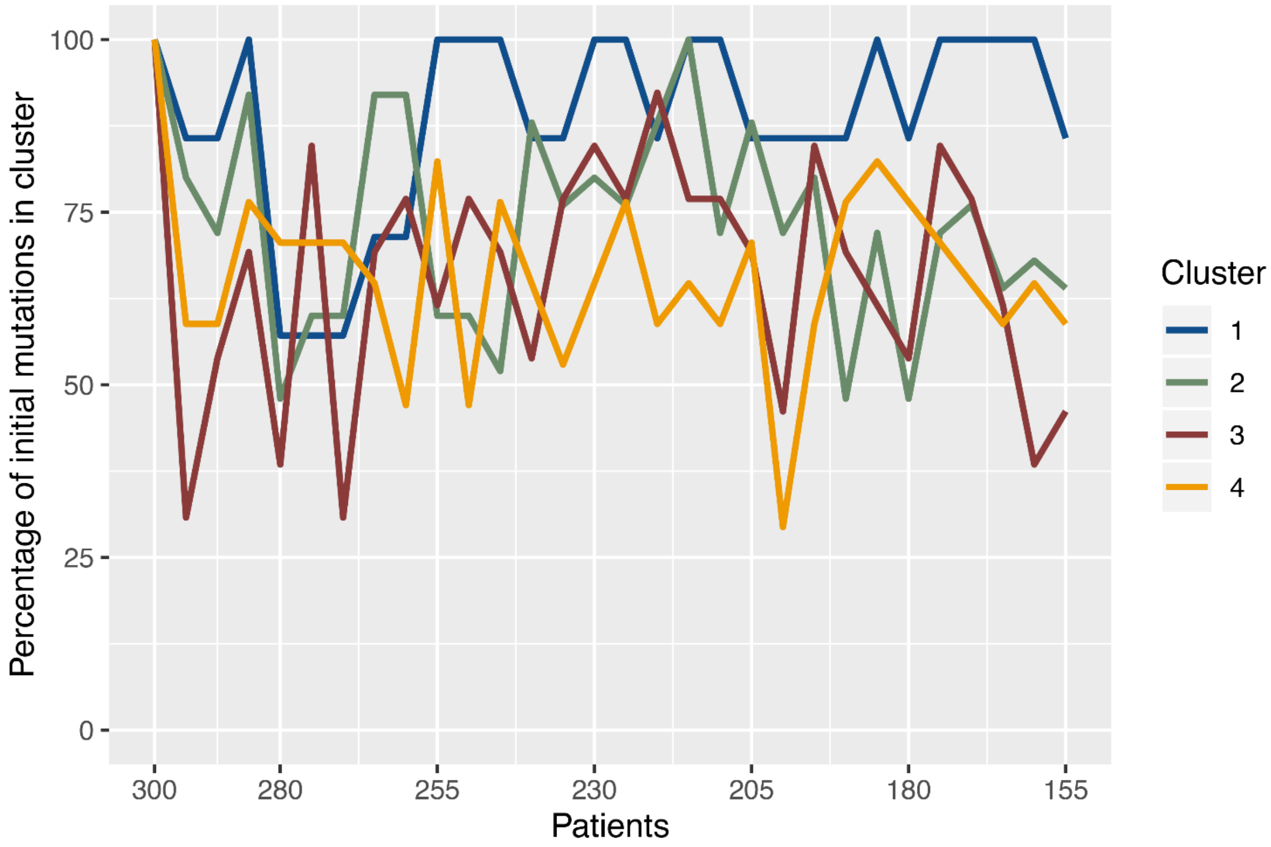

Figure S3: Step-wise subsampling of the patient cohort to ensure a high stability of extracted signatures defining the patient clusters. For each step the patient cohort was reduced by 5 randomly chosen patients and the resulting percentage of genes assigned to their original signature was calculated. Each NMF analysis was performed using a fixed factorization rank (k=4). Even after the removal of almost half the patients, ¾ of the signatures retained over 60% of their originally defining genetic events.

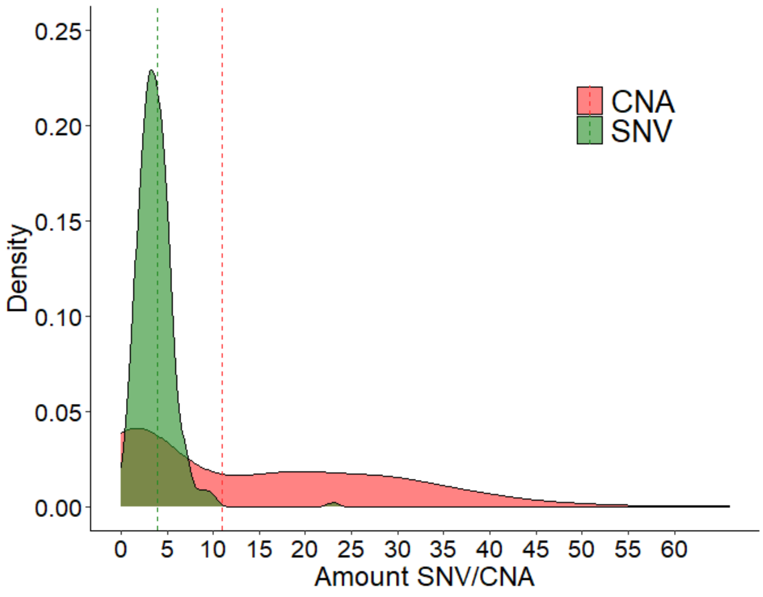

Figure S4: Distribution of SNV/CNA per patient in complete cohort. Dashed lines mark the median alteration amount.

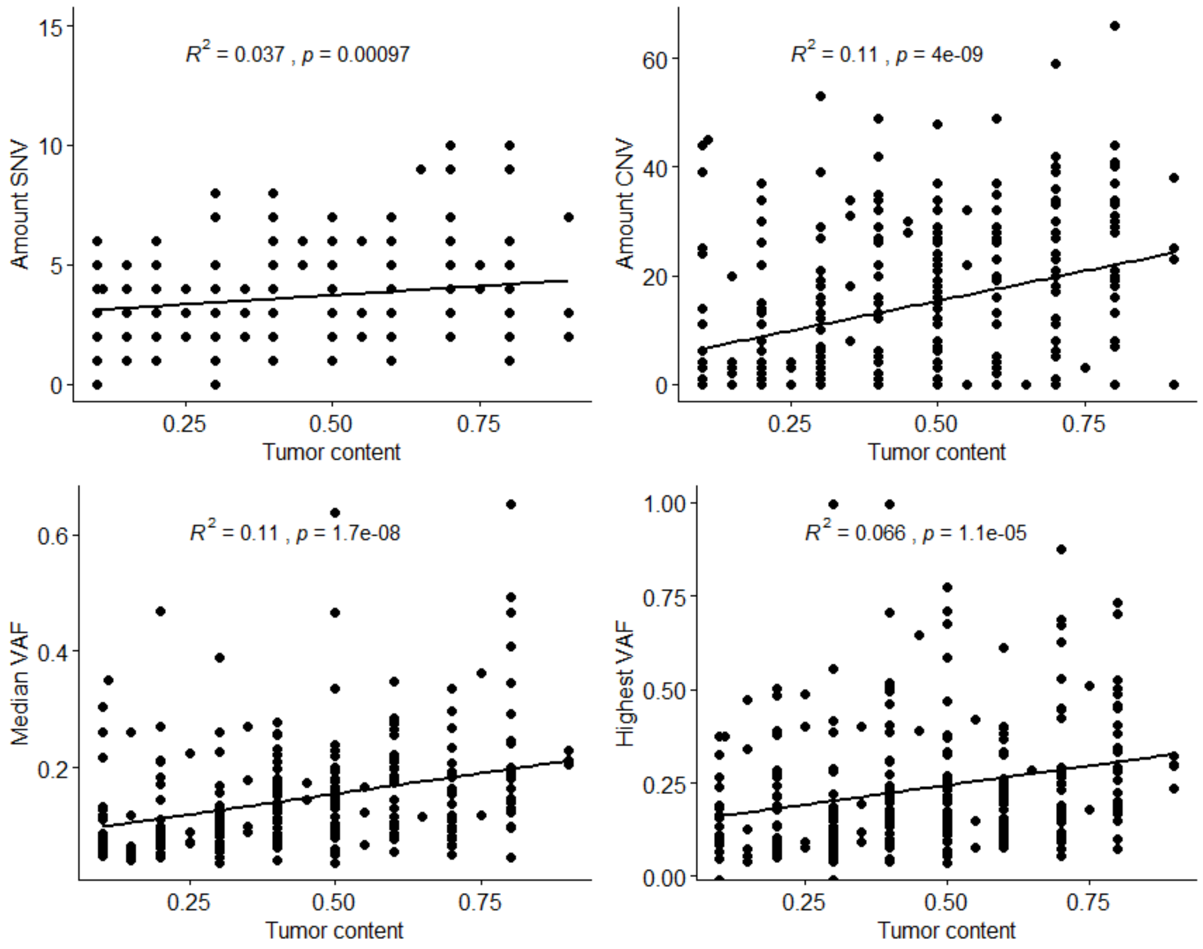

Figure S5: Correlation of morphological estimation of tumor content with number of SNVs, number of CNAs, mean VAF and highest VAF per patient No significant correlation between tumor content and amount of alteration or VAF were observed.

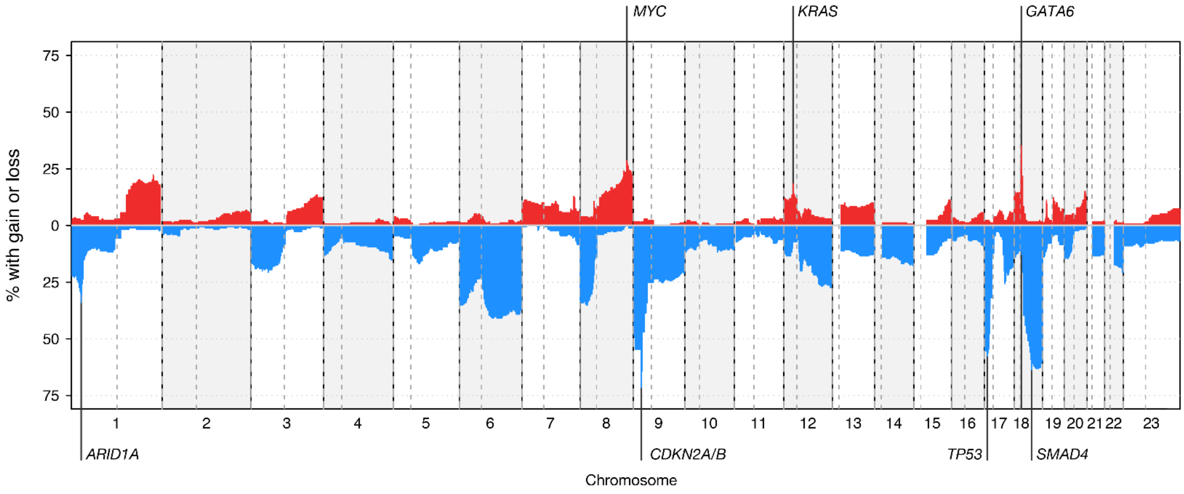

Figure S6: Genome wide CNAs in 171 patients with complete copy number information. Example genes for most frequently altered regions are highlighted.

SNV

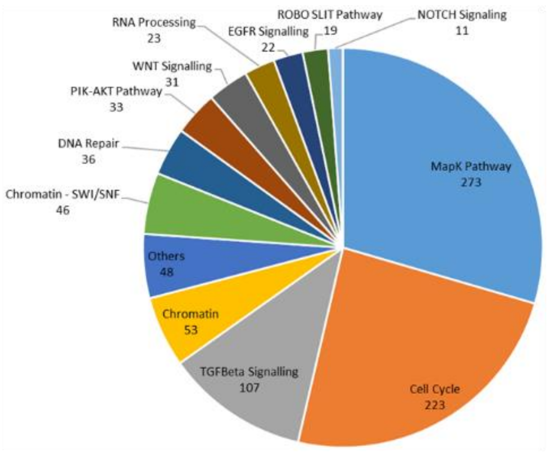

CNA

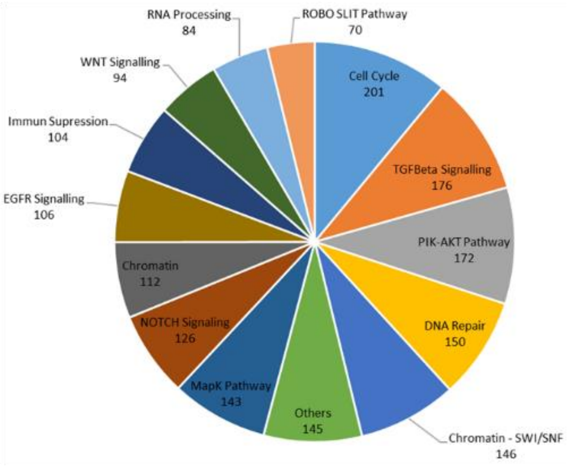

Combined

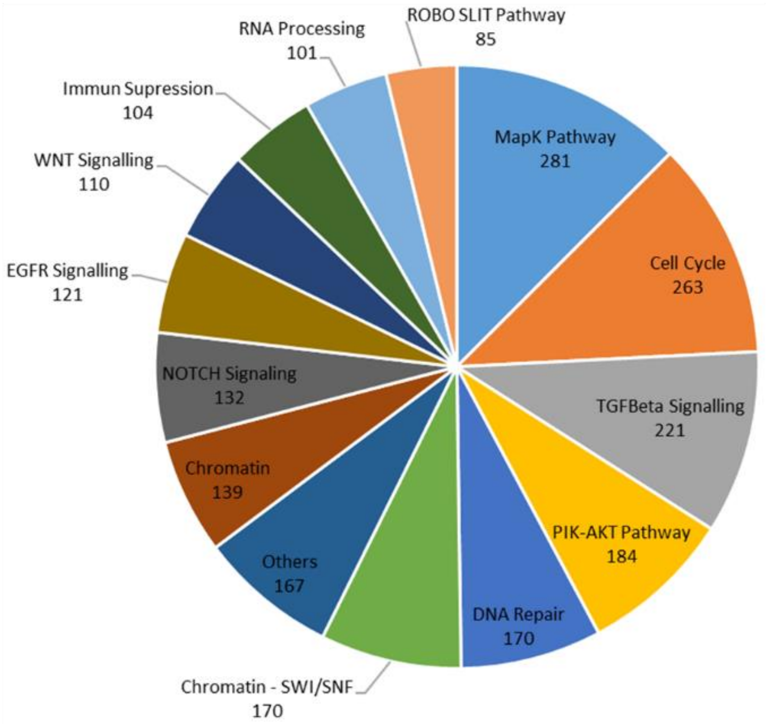

Figure S7: Percentage of patients with genes altered (either A) SNV or B) CNV or C) both) in major signaling pathways covered by our NGS panel. Pathway affiliations according to KEGG and Bailey et al. (see Table S7).[3]

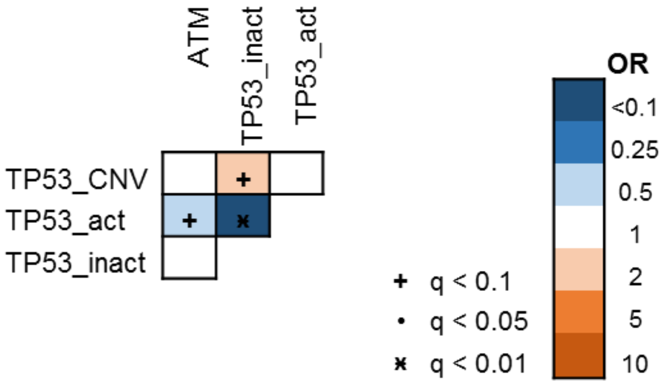

Figure S8: Co-occurrence and mutual exclusivity test for *TP53* mutations. Significance levels of q-values (multiple testing corrected) are shown with symbols, odds ratio with colors (blue show different levels of mutual exclusivity, orange show different levels of co-mutation). For a list of all *TP53* mutations with functional consequences see Supplemental Table S8.

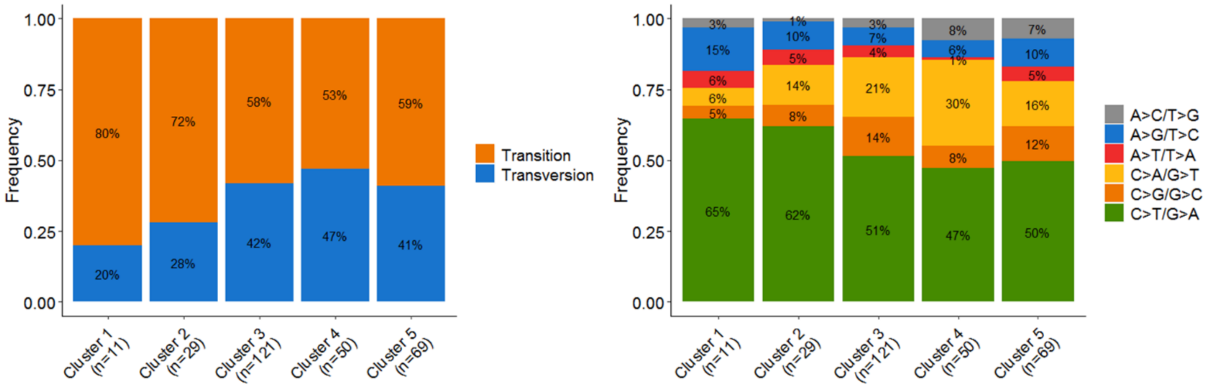

Figure S9: Base change signature of the five patient clusters, comparing both the proportion of transitions vs transversions as well as the individual base changes within each cluster. For the analysis all non-synonymous SNVs from each patient of a cluster were included.

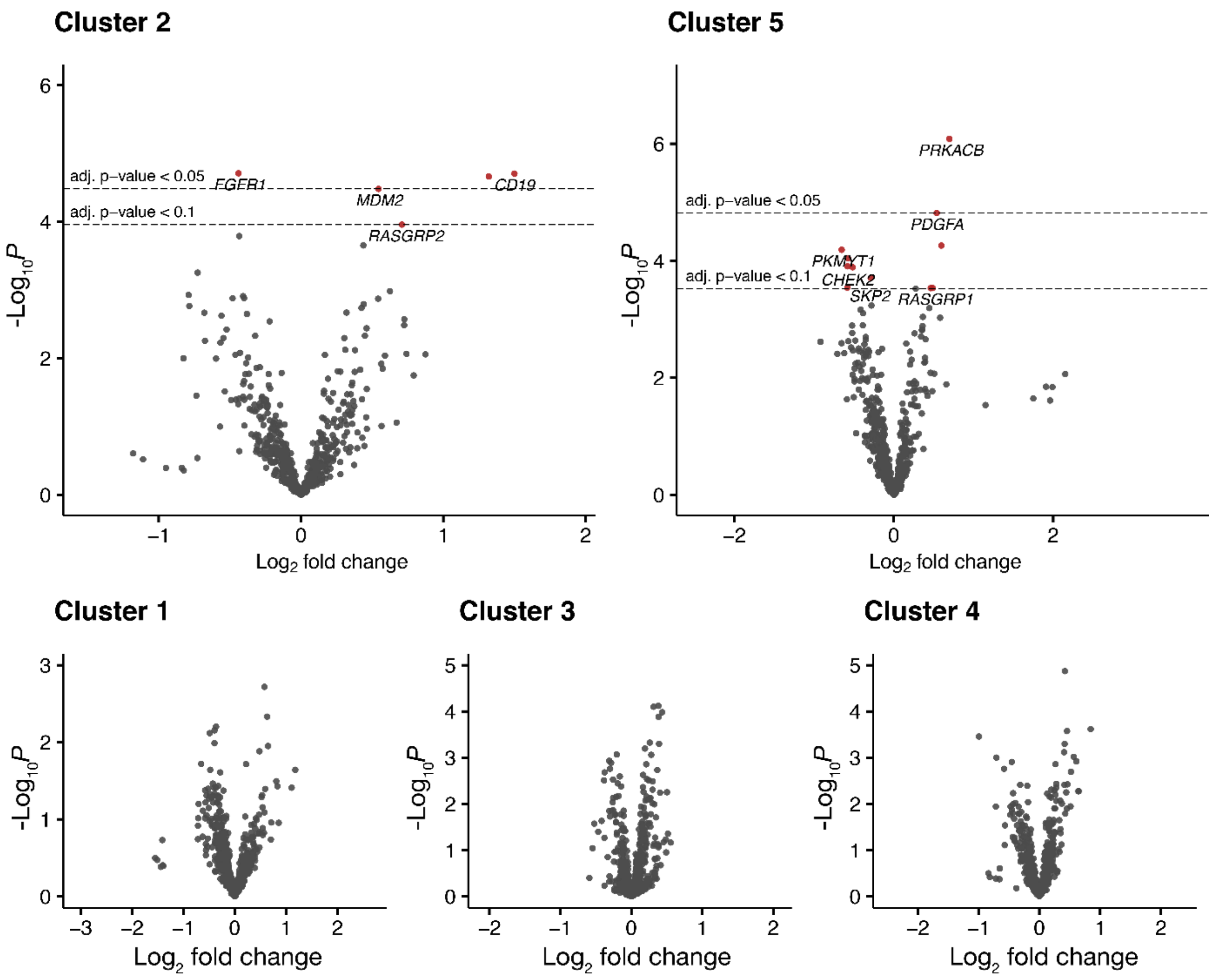

Figure S10: Volcano plots depicting differential expression analyses comparing gene expression data obtained from the Nanostring PanCancerPanel of each cluster with the rest of the cohort. Horizontal lines show significance levels of p-values (multiple testing adjusted with Benjamini-Hochberg). The 50 variants with the lowest p-value are labelled.

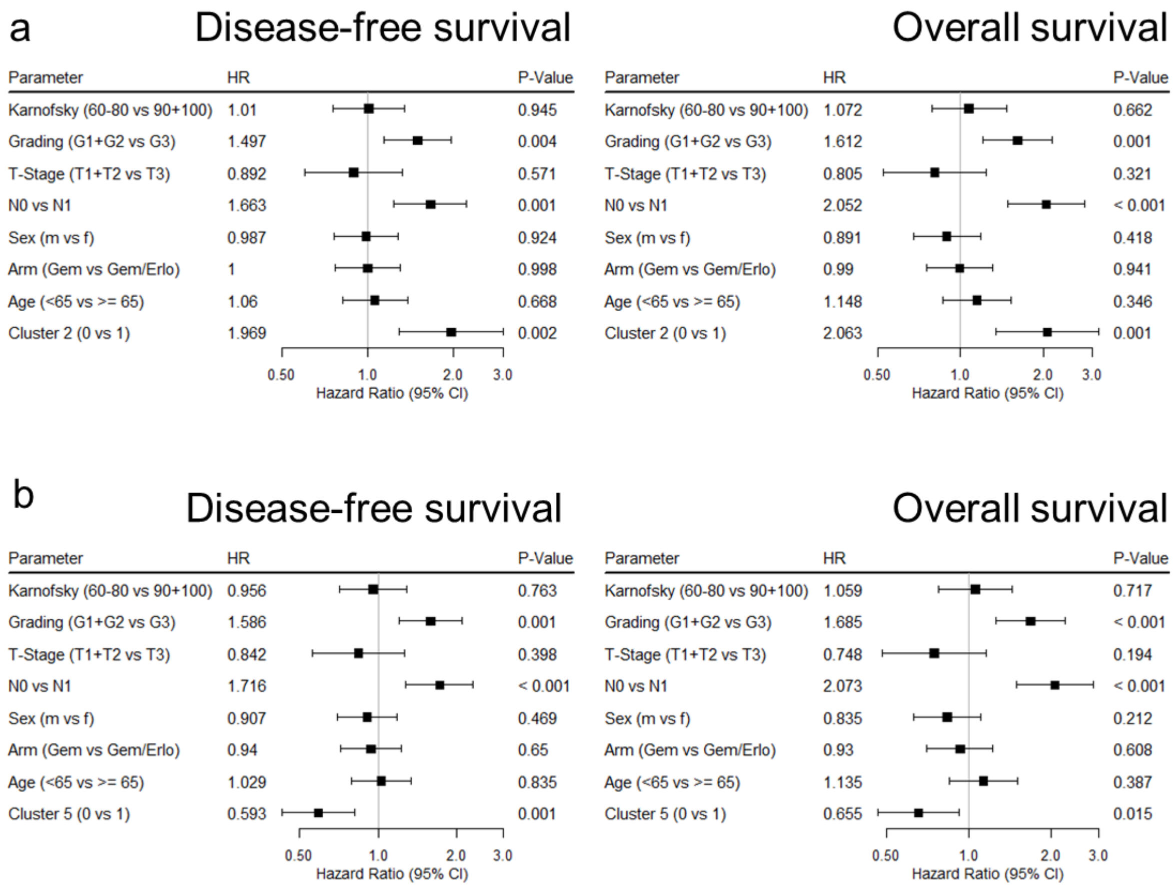

Figure S11: Forest plot of multivariate cox-hazard analysis showing the significant impact on both DFS and OS of **a)** cluster 2 and **b)** cluster 5.

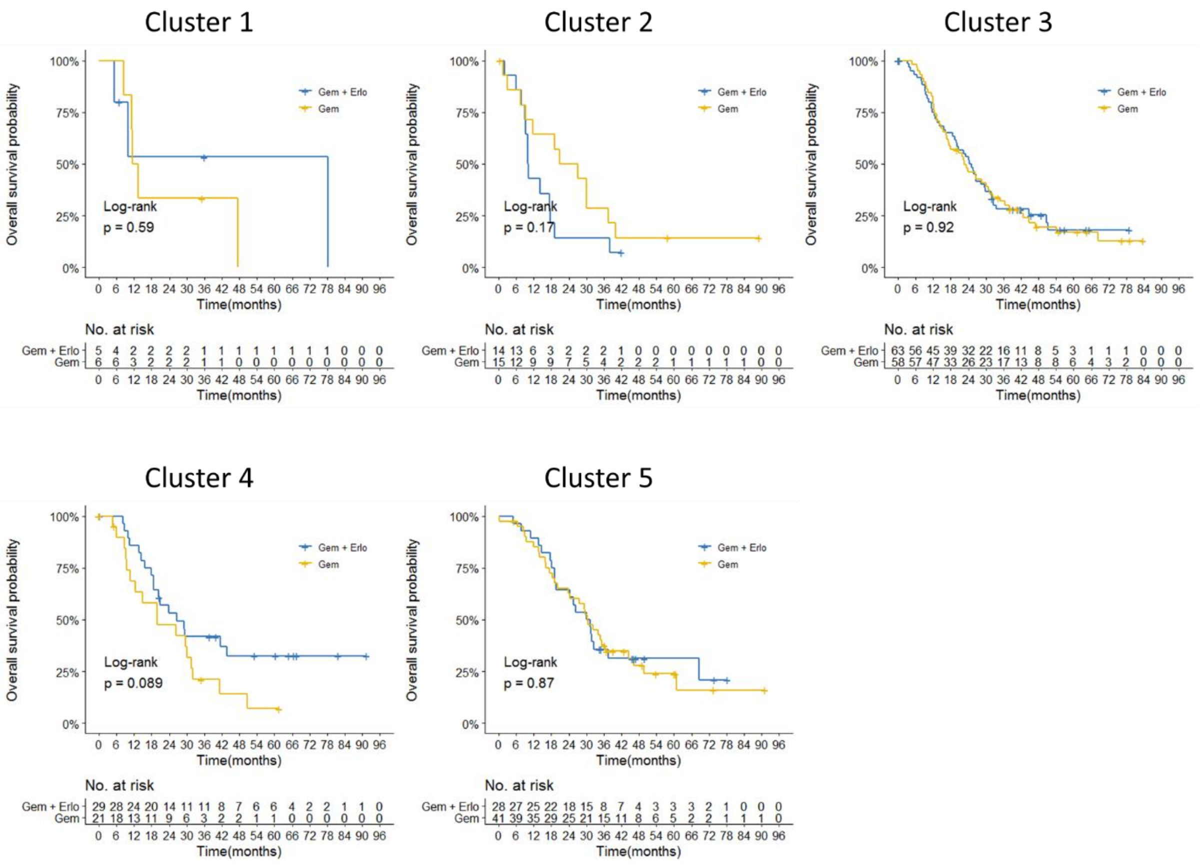

Figure S12: Overall survival according to both treatment arms of the CONKO-005 trial within each of the five patient clusters.

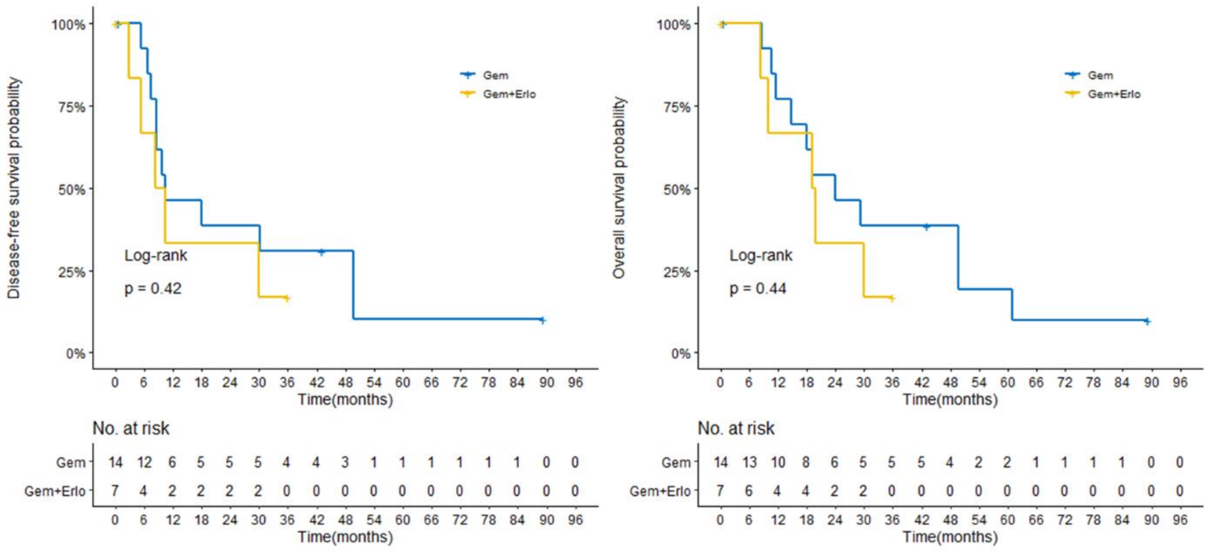

Figure S13: DFS and OS of patients with *EGFR* mutations (n=4) and amplifications (n=17) comparing both treatment arms.

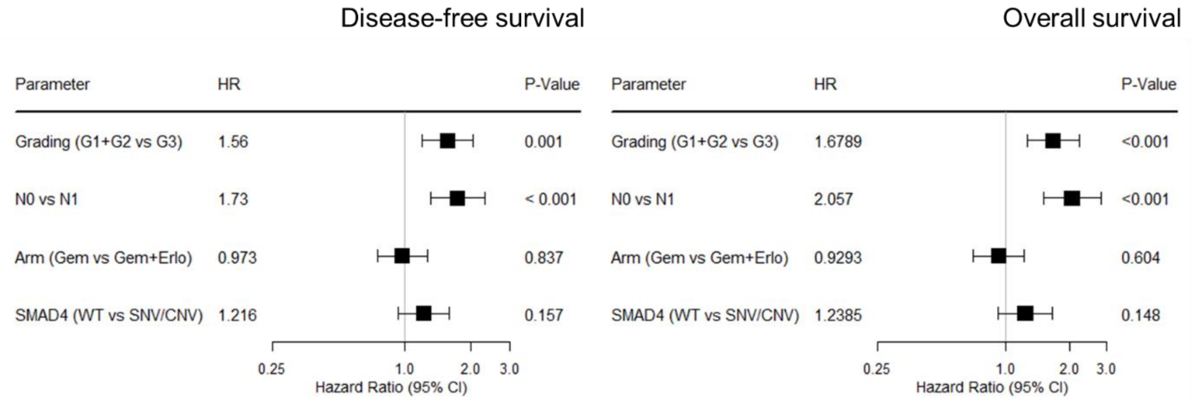

Figure S14: Forest plot of multivariate Cox-hazard analysis showing the impact of treatment arm and *SMAD4* status on OS and DFS.

Tables

Table S1: Morphological estimation of tumor content of all 293 samples.

See additional excel file

Table S2: List of genes included in the custom SureSelectXT panel (Agilent). Probes covering the full-length coding region of all 67 genes were included and subsequently used for SNV detection.

Gene	Gene	Gene	Gene
ACVR1B	*GLI3*	*PIK3R5*	*SMARCA4*
ACVR2A	*GNAS*	*PLCG2*	*TGFBR1*
APC	*HUWE1*	*PLXNB2*	*TGFBR2*
ARID1A	*KDM6A*	*PMS2*	*TLE4*
ARID2	*KMT2C*	*PREX2*	*TP53*
ATM	*KMT2D*	*PRKCG*	*TP53BP2*
BCORL1	*KRAS*	*PRSS1*	*U2AF1*
BRAF	*MAP2K4*	*RBM10*	
BRCA1	*MAP2K7*	*RBM6*	
BRCA2	*MAPT*	*RNF43*	
CALD1	*MARK2*	*ROBO1*	
CAMK2B	*MSH2*	*ROBO2*	
CDKN2A	*MYC*	*RPS6KA2*	
EGF	*MYCBP2*	*SETD2*	
EGFR	*NF2*	*SF3A1*	
ERBB2	*NRG1*	*SF3B1*	
ERBB3	*PALB2*	*SLIT2*	
ErbB4	*PBRM1*	*SMAD3*	
FBXW7	*PIK3CA*	*SMAD4*	
GATA6	*PIK3CG*	*SMARCA2*	

Table S3: List of genes with gene specific probes (3-6 per gene) included in the custom SureSelectXT panel (Agilent) and subsequently used for enabled CNA detection.

Genes	Genes	Genes	Genes	Genes
ACVR1B	*CDKN2A*	*KMT2D*	*PDCD1*	*ROBO2*
ACVR2A	*CDKN2B*	*KRAS*	*PDCD1LG2*	*RPA1*
AKT1	*EGF*	*MAP2K4*	*PDGFRA*	*RPS6KA2*
APC	*EGFR*	*MAP2K7*	*PIK3CA*	*SETD2*
ARID1A	*ERBB2*	*MAPT*	*PIK3CG*	*SF3A1*
ARID1B	*ERBB3*	*MARK2*	*PIK3R1*	*SF3B1*
ARID2	*ERBB4*	*MET*	*PIK3R3*	*SLIT2*
ATM	*FBXW7*	*MIB1*	*PIK3R5*	*SMAD3*
BCORL1	*FGFR1*	*MLH1*	*PLCG2*	*SMAD4*
BRAF	*FGFR2*	*MSH2*	*PLXNB2*	*SMARCA2*
BRCA1	*FGFR3*	*MYB*	*PMS2*	*SMARCA4*
BRCA2	*GATA6*	*MYC*	*PREX2*	*SMARCB1*
CALD1	*GLI3*	*MYCBP2*	*PRKCG*	*SOX9*
CAMK2B	*GNAS*	*NCOR1*	*PRSS1*	*STK11*
CASP8	*HUWE1*	*NF2*	*PTEN*	*TGFBR1*
CCND2	*JAK2*	*NOTCH1*	*RB1*	*TGFBR2*
CCNE1	*KDM6A*	*NOV*	*RBM10*	*TLE4*
CD274	*KIT*	*NRG1*	*RBM6*	*TP53*
CDH1	*KMT2A*	*PALB2*	*RNF43*	*TP53BP2*
CDK6	*KMT2C*	*PBRM1*	*ROBO1*	*U2AF1*

Table S4: List of genes and positions included in the customized version of the PanCancer Pathways Panel (NanoString Technologies).

Customer Identifier	Accession	Position	Target Sequence
AURKA	NM_003600.2	406-505	AGCTCCAGTTGGAGGTCCAAAACGTGTTCTCGTGACTCAGCAATTTCC<br>TTGTCAGAATCCATTACCTGTAAATAGTGGCCAGGCTCAGCGGGTCTTGTGT
CALML4	NM_033429.2	3431-3530	CTCCTCTTCACGGACTCCCTGCTCATTAAGGGATTCAGTGGTCCAGAGTCT<br>AAGATCCTATTAAGTGTTTGATTCAAACCTCTACCCGAGGAAGGGCTGT
CD274	NM_014143.3	50-149	AGCTTCCCGAGGCTCCGCACCAGCCGCGCTTCTGTCCGCCTGCAGGGCA<br>TTCCAGAAAGATGAGGATATTTGCTGTCTTTATATTCATGACCTACTGGCA
CELA3B	XM_011541132.1	877-976	CAAGGCACCAGAGTCCCGGACTTAGGACCACTGTGTGTGATTCACCGTG<br>AACACTGGCATATTAAAGGCTCTGAGAAGTCCTGTAGGAAAGGACAATTTG
CIITA	NM_000246.3	471-570	GCCTGAGCAAGGACATTTTCAAGCACATAGGACCAGATGAAGTGATCG<br>GTGAGAGTATGGAGATGCCAGCAGAAGTTGGGCAGAAAAGTCAGAAAAGACC
CLPS	NM_001252598.1	173-272	TGAGCGTGGCCTGACCTGTGAGGGAGACAAGACCATCGTGGGCTCCAT<br>CACCAACACCAACTTTGGCATCTGCCATGACGCTGGACGCTCCAAGCAGTGA
CPA2	NM_001869.2	233-332	GTCAACGTCCAGGCAGTCAAAGTGTTCTTGGAGTCCCAGGGAATTGCCT<br>ATTCCATCATGATTGAAGACGTGCAGGTCCTGTTGGACAAAGAGAATGAAG
CBP1	NM_001871.2	867-966	GAGACCAAGGCCCTGGCTGATTTCATCCGCAACAAACTCTCTTCCATCAA<br>GGCATATCTGACAATCCACTCGTACTCCCAAATGATGATCTACCCTTACT
CTRB1	NM_001906.4	694-793	CTGGACCCTGGTGGGCATTGTGTCCTGGGGCAGCGACACCTGCTCCAC<br>CTCCAGCCCTGGCGTGTACGCCCGTGTCACCAAGCTCATACCTTGGGTGCAG
ErbB3	NM_001005915.1	421-520	CCTGCAGTGGATTCGAGAAGTGACAGGCTATGTCCTCGTGGCCATGAA<br>TGAATTCTCTACTCTACCATTGCCCAACCTCCGCGTGGTGCGAGGGACCCAG
ERBB4	NM_001042599.1	7301-7400	CTAGGATAGATCCAGAACCACAAAGCATCTGCACCACAAAAGGTGTT<br>AGACTACCAAGCAGCTCCTGGTTTTCTGCATAGTATTAGTAGCACAGCTTAGG
HNF4G	NM_004133.3	2196-2295	CTATAGGTGGAAAGTCTGTGTCAGCCAAAGCCATGTCACTCTGAGAGAAC<br>TGATTCTGAGGACAAGTTAGCCATCTACCACCCATGTCCCTTATTTGGTC
IGLC3	ENST00000390325.1	177-276	AAACAAAGCAACAACAAGTACGCGGCCAGCAGCTACCTGAGCCTGACGC<br>CTGAGCAGTGGAAGTCCCACAGAAGCTACAGCTGCCAGGTCACGCATGAAG
IGLL5	NM_001178126.1	959-1058	CAGGATCCCAGGGGAGGGGTCTCTCTCCCCATCCCAAGTCATCCAGCCC<br>TTCTCCCTGCACTCATGAAACCCCAATAAATATCCTCATTGACAACCAGAA
ITPKB	NM_002221.3	79-178	GGTTTGCGCCTCTGGGCATGTAGTCTACACAGGACCTGAGAATCTGAGA<br>AACTGCAGCCGCACGGTTGTTTATGGAGCTTTGGGCGGGGGCTGAGCCCGC
LRRC66	NM_001024611.1	1661-1760	GCACATTCAGTGGGCGTCTCTTCTGTAGCTGGCACGTCTCACGCTGTCT<br>CTGGCTCAAGCCGTTATGATTCCAATGAATTAGACCCTTCCCTCTCCGGAG
MAP2K7	NM_145185.2	496-595	ACGTCATTGCCGTTAAGCAAATGCGGCGCTCCGGGAACAAGGAGGAGA<br>ACAAGCGCATCCTCATGGACCTGGATGTGGTGCTGAAGAGCCACGACTGCCC
NPSR1	NM_207172.1	1007-1106	TGACATTTTGGACAATTTCAACCTCCTTCCAGACACCCAGGAGCGTTTCT<br>ATGCCTCTGTGATCATTCAGAACCTGCCAGCATTGAATAGTGCCATCAAC
PDCD1LG2	NM_025239.3	236-335	TGTGGAGCTGTGGCAAGTCCTCATATCAAATACAGAACATGATCTTCC<br>TCCTGCTAATGTTGAGCCTGGAATTGCAGCTTCACCAGATAGCAGCTTTATT
PRSS1	NM_002769.4	361-460	CACGTGCAGTAATCAACGCCCGCGTGTCCACCATCTCTCTGCCCACCGC<br>CCCTCCAGCCACTGGCACGAAGTGCCTCATCTCTGGCTGGGGCAACACTGC
S100A4	NM_002961.2	264-363	CAGGGACAACGAGGTGGACTTCCAAGAGTACTGTGTCTTCCTGTCCTG<br>CATCGCCATGATGTGTAACGAATTCTTTGAAGGCTTCCCAGATAAGCAGCCC
SMPD3	NM_018667.3	473-572	CTGTGTGTCCTGGGCCCTTATCTTTCCATGCTACTGGCTGGTGGACCG<br>GCTCGCTGCCTCCTTCATACCCACCACCTACGAGAAGCGCCAGCGGGCAGAC
STAT6	NM_003153.3	2031-2130	AGAACATCCAGCCATTCTCTGCCAAAGACCTGTCCATTCGCTCACTGG<br>GGGACCGAATCCGGGATCTTGCTCAGCTCAAAAATCTCTATCCCAAGAAGCC
TGFA	NM_003236.2	781-880	TGCCACAGACCTTCCTACTTGGCCTGTAATCACCTGTGCAGCCTTTTGT<br>GGGCCTTCAAAACTCTGTCAAGAACTCCGTCTGCTTGGGGTTATTCAGTGT
TGFBR1	NM_004612.2	1256-1355	GAATCCTTCAAACGTGCTGACATCTATGCAATGGGCTTAGTATTCTGG<br>GAAATTGCTCGACGATGTTCCATTGGTGGAATTCATGAAGATTACCAACTGC
ZFP36L1	NM_004926.2	2767-2866	CCACGTGCCCATCTCAAGACATTCCACTCACAGATTTGAGGTTCTGGAT<br>TCCAGGTCTGGAGTTTTCCAATGTTAATGTAAACAGAACTGGCACACACAC
ZNF217_2	NM_006526.2	5095-5194	AACACATTTGCAAGATGATTGACTCAATCTTTGCCTAATCCAATGAGTGT<br>TACAGAGAGCTTGCTGTGACTAGAACCATAAATCTTAAAGGGGGTATGTG
ZNF217_1	NM_006526.2	515-614	AAACATGTCTTAATGCAACACCGGCCTACCCTCTGTGAACCAGCAGTTC<br>TTCGGGTTGAAGCAGAGTATCTCAGTCCGCTTGATAAAAGTCAAGTGCGAA

Table S5: 1086 SNVs detected by targeted sequencing in 293 PDAC patients.

See additional excel file

Table S6: 4157 CNAs detected by targeted sequencing and MLPA analyses in 293 PDAC patients.

See additional excel file

Table S7: Assignment of genes to their respective pathway annotation. Pathway affiliations according to KEGG and Bailey et al.[3]

Pathway	Genes
**Cell Cycle**	*CDKN2A, TP53, TP53BP2, MYC, CCND2, CCNE1, CDK6, CDKN2B, RB1*
**Chromatin**	*KDM6A, KMT2C, KMT2D, SETD2, KMT2A, NCOR1, SMARCB1, ARID1A, SMARCA4, PBRM1, ARID2, SMARCA2, ARID1B*
**DNA Repair**	*BRCA1, BRCA2, ATM, PALB2, MSH2, PMS2, MLH1, RPA1*
**EGFR Signaling**	*EGF, EGFR, ERBB2, ERBB3, ERBB4, NRG1, Camk2b*
**Immune Suppression**	*CD274, JAK2, PDCD1, PDCD1LG2*
**MAPK Pathway**	*MAP2K4, MAP2K7, MAPT, BRAF, KRAS, RPS6KA2, FGFR1, FGFR2, FGFR3, KIT, MET, PDGFRA*
**NOTCH Signaling**	*NF2, BCORL1, FBXW7, MIB1, NOTCH1, NOV*
**PIK-AKT Pathway**	*PIK3CA, PIK3CG, PIK3R5, PREX2, AKT1, MYB, PIK3R1, PIK3R3, PTEN, STK11*
**RNA Processing**	*RBM10, SF3B1, U2AF1, RBM6, SF3A1*
**ROBO SLIT Pathway**	*ROBO1, ROBO2, SLIT2, MYCBP2*
**TGFbeta Signaling**	*SMAD3, SMAD4, TGFBR1, TGFBR2, ACVR1B, ACVR2A*
**WNT Signaling**	*RNF43, TLE4, APC*
**Others**	*SOX9, GATA6, GLI3, MARK2, PLXNB2, CALD1, GNAS, HUWE1, PLCG2, PRKCG, PRSS1, CASP8, CDH1*

Table S8: List of *TP53* mutations with functional consequences (activating vs inactivating)[20].

See additional excel file

Table S9: Clinical baseline characteristics of patients within the five patient clusters.

Characteristics		Cluster 1	Cluster 2	Cluster 3	Cluster 4	Cluster 5
		(n = 11)	(n = 29)	(n = 121)	(n = 50)	(n = 69)
**Age (years)**						
	Median	68	63	64	64,5	64
	range	46 - 75	45-80	35-82	44-80	24-76
**Sex**						
	male - no. (%)	8 (73%)	18 (62%)	62 (51%)	22 (44%)	43 (62%)
	female - no. (%)	3 (27%)	11 (38%)	59 (49%)	28 (56%)	26 (38%)
**Arm**						
	Gemcitabine – no. (%)	6 (55%)	15 (52%)	58 (48%)	21 (42%)	41 (59%)
	Gemcitabine + Erlotnib - no. (%)	5 (45%)	14 (48%)	63 (53%)	29 (58%)	28 (41%)
**Karnofsky**						
	60 - no. (%)	0 (0%)	1 (3%)	0 (0%)	0 (0%)	0 (0%)
	70 - no. (%)	1 (9%)	2 (7%)	2 (2%)	2 (4%)	2 (3%)
	80 - no. (%)	3 (27%)	5 (17%)	33 (27%)	16 (32%)	14 (20%)
	90 - no. (%)	3 (27%)	12 (41%)	47 (39%)	16 (32%)	28 (41%)
	100 - no. (%)	4 (36%)	9 (31%)	39 (32%)	16 (32%)	25 (36%)
**Grading**						
	G1 - no. (%)	1 (9%)	0 (0%)	1 (1%)	2 (4%)	2 (3%)
	G2 - no. (%)	5 (45%)	14 (48%)	77 (64%)	35 (70%)	38 (55%)
	G3 - no. (%)	5 (45%)	13 (45%)	38 (31%)	12 (24%)	27 (39%)
	unknown - no. (%)	0 (0%)	2 (7%)	5 (4%)	1 (2%)	2 (3%)
**T-Stage**						
	T1 - no. (%)	1 (9%)	0 (0%)	0 (0%)	5 (10%)	2 (3%)
	T2 - no. (%)	1 (9%)	4 (14%)	11 (9%)	4 (8%)	9 (13%)
	T3 - no. (%)	9 (82%)	25 (86%)	107 (88%)	41 (82%)	58 (84%)
	T4 - no. (%)	0 (0%)	0 (0%)	3 (2%)	0 (0%)	0 (0%)
**N-Stage**						
	N0 - no. (%)	6 (55%)	9 (31%)	42 (35%)	16 (32%)	26 (38%)
	N1 - no. (%)	5 (45%)	20 (69%)	79 (65%)	34 (68%)	43 (62%)
**Postoperative CA 19-9 (kU/L)**						
	Median (range)	13 (1-1976)	31 (2-1000)	19 (1-2573)	11 (1-405)	12 (1-77)
	<= 100 - no. (%)	9 (82%)	25 (86%)	99 (82%)	34 (68%)	44 (64%)
	101-500 - no. (%)	1 (9%)	1 (3%)	10 (8%)	7 (14%)	9 (13%)
	> 500 - no. (%)	1 (9%)	1 (3%)	4 (3%)	4 (8%)	1 (1%)
	unknown - no. (%)	0 (0%)	2 (7%)	8 (7%)	5 (10%)	15 (22%)

Table S10: Summary of clinical and genetic characteristics of patient clusters.

	**Cluster 1**	**Cluster 2**	**Cluster 3**	**Cluster 4**	**Cluster 5**
**Genetic alteration**	Mutations ↑ ERBB Pathway ↑	Deletions Tumor Suppressor ↑	Deletions PDAC genes ↑	Deletions *CDKN2A* ↓ *SMAD4* mutations ↑	Deletions PDAC genes ↓ *KRAS* mutations ↓
**Base Change**	Transversion ↓	-	-	C>A/G>T ↑	-
**Survival**	-	DFS ↓ OS ↓	-		DFS ↑ OS ↑
**Differential Expression**	-	PI3K-/AKT Pathway ↑	-	-	MAPK Pathway ↑ *PTEN* ↑ Cell Cycle control ↓
**Erlotinib effect**	-	-	-	OS ↑	-

Table S11: Results of the differential expression analysis comparing *SMAD4* altered (*SMAD4*^{alt}) patients with *SMAD4* wild-type (*SMAD4*^{WT}) patients. *SMAD4* normal patients were used as baseline.

See additional excel file

Table S12: Integration of *MAPK9* gene expression data with *SMAD4* genetic alteration status. *MAPK9* expression levels were dichotomized into high and low expression based on 230 PDAC patients analyzed by NanoString. No *SMAD4* status was available from eight of these 230 patients.

	SMAD4^{alt}	***SMAD4***^{WT}	total
MAPK9^{low}	n=91	n=22	113
MAPK9^{high}	n=62	n=47	109
total	n=153	n=69	

References

1. Sinn M, Bahra M, Liersch T, et al: CONKO-005: Adjuvant Chemotherapy With Gemcitabine Plus Erlotinib Versus Gemcitabine Alone in Patients After R0 Resection of Pancreatic Cancer: A Multicenter Randomized Phase III Trial. Journal of Clinical Oncology:JCO.2017.72.6463, 2017
2. Waddell N, Pajic M, Patch A-M, et al: Whole genomes redefine the mutational landscape of pancreatic cancer. Nature 518:495-501, 2015
3. Bailey P, Chang DK, Nones K, et al: Genomic analyses identify molecular subtypes of pancreatic cancer. Nature 531:47-52, 2016
4. Vincent A, Herman J, Schulick R, et al: Pancreatic cancer. Lancet (London, England) 378:607-620, 2011
5. Sausen M, Phallen J, Adleff V, et al: Clinical implications of genomic alterations in the tumour and circulation of pancreatic cancer patients. Nat Commun, 2015
6. Kosmidis C, Sapalidis K, Kotidis E, et al: Pancreatic cancer from bench to bedside: molecular pathways and treatment options. Annals of Translational Medicine 4, 2016
7. GeneDx: Pancreatic Cancer Panel. https://www.genedx.com/test-catalog/available-tests/pancreatic-cancer-panel/, 2016
8. Seki M, Kimura S, Isobe T, et al: Recurrent SPI1 (PU.1) fusions in high-risk pediatric T cell acute lymphoblastic leukemia. Nature Genetics 49:1274, 2017
9. Shiraishi Y, Sato Y, Chiba K, et al: An empirical Bayesian framework for somatic mutation detection from cancer genome sequencing data. Nucleic Acids Research 41:e89-e89, 2013
10. Robinson JT, Thorvaldsdóttir H, Winckler W, et al: Integrative genomics viewer. Nature Biotechnology 29:24-26, 2011
11. Christen F, Hoyer K, Yoshida K, et al: Genomic landscape and clonal evolution of acute myeloid leukemia with t(8;21): an international study on 331 patients. Blood 133:1140-1151, 2019
12. Arends CM, Galan-Sousa J, Hoyer K, et al: Hematopoietic lineage distribution and evolutionary dynamics of clonal hematopoiesis. Leukemia 32:1908-1919, 2018
13. Yoshizato T, Nannya Y, Atsuta Y, et al: Genetic abnormalities in myelodysplasia and secondary acute myeloid leukemia: impact on outcome of stem cell transplantation. Blood 129:2347-2358, 2017
14. Benjamini Y, Hochberg Y: Controlling the False Discovery Rate: A Practical and Powerful Approach to Multiple Testing. Journal of the Royal Statistical Society. Series B (Methodological) 57:289-300, 1995
15. Teixeira VH, Pipinikas CP, Pennycuick A, et al: Deciphering the genomic, epigenomic, and transcriptomic landscapes of pre-invasive lung cancer lesions. Nature Medicine 25:517-525, 2019
16. Mylonas E, Yoshida K, Frick M, et al: Single-cell analysis based dissection of clonality in myelofibrosis. Nature Communications 11:73, 2020
17. Hüebschmann D, Kurzawa N, Steinhauser S, et al: Deciphering programs of transcriptional regulation by combined deconvolution of multiple omics layers. bioRxiv:199547, 2017
18. Gao J, Aksoy BA, Dogrusoz U, et al: Integrative analysis of complex cancer genomics and clinical profiles using the cBioPortal. Sci Signal 6:pl1, 2013
19. Cerami E, Gao J, Dogrusoz U, et al: The cBio Cancer Genomics Portal: An Open Platform for Exploring Multidimensional Cancer Genomics Data. Cancer Discovery 2:401-404, 2012
20. Muller Patricia AJ, Vousden Karen H: Mutant p53 in Cancer: New Functions and Therapeutic Opportunities. Cancer Cell 25:304-317, 2014
